# Proteogenomic analysis of enriched HGSOC tumor epithelium identifies prognostic signatures and therapeutic vulnerabilities

**DOI:** 10.1038/s41698-024-00519-8

**Published:** 2024-03-13

**Authors:** Nicholas W. Bateman, Tamara Abulez, Anthony R. Soltis, Andrew McPherson, Seongmin Choi, Dale W. Garsed, Ahwan Pandey, Chunqiao Tian, Brian L. Hood, Kelly A. Conrads, Pang-ning Teng, Julie Oliver, Glenn Gist, Dave Mitchell, Tracy J. Litzi, Christopher M. Tarney, Barbara A. Crothers, Paulette Mhawech-Fauceglia, Clifton L. Dalgard, Matthew D. Wilkerson, Mariaelena Pierobon, Emanuel F. Petricoin, Chunhua Yan, Daoud Meerzaman, Clara Bodelon, Nicolas Wentzensen, Jerry S. H. Lee, The APOLLO Research Network, Sasha C. Makohon-Moore, Waleed Barakat, Xijun Zhang, Allison Hunt, Wei Ao, Stacey L. Lytle-Gabbin, Yovanni Casablanca, Chad A. Hamilton, Miranda Newell, Justin Wells, Gauthaman Sukumar, Dagmar Bacikova, John Freyman, David E. Cohn, Andrew Berchuck, Laura Havrilesky, Linda Duska, Adekunle Odunsi, Anil Sood, James Brenton, Evis Sala, Christina Annunziata, Oliver Dorigo, Brad Nelson, Dawn R. Cochrane, Kathleen Moore, Elisa Baldelli, Qing-rong Chen, Ying Hu, Sian Fereday, Nadia Traficante, Anna DeFazio, Ellen L. Goode, David G. Huntsman, Sohrab Shah, Craig D. Shriver, Neil T. Phippen, Kathleen M. Darcy, David D. L. Bowtell, Thomas P. Conrads, G. Larry Maxwell

**Affiliations:** 1 Gynecologic Cancer Center of Excellence, Gynecologic Surgery and Obstetrics, Uniformed Services University of the Health Sciences, Bethesda, MD USA; 2 The Henry M. Jackson Foundation for the Advancement of Military Medicine Inc, Bethesda, MD USA; 3 The John P. Murtha Cancer Center Research Program, Department of Surgery, Uniformed Services University and Walter Reed National Military Medical Center, Bethesda, MD USA; 4 The American Genome Center, Collaborative Health Initiative Research Program, Department of Anatomy, Physiology and Genetics, Uniformed Services University of the Health Sciences, Bethesda, MD USA; 5 Department of Computational Oncology, Memorial Sloan Kettering Cancer Center, Manhattan, NY USA; 6 Peter MacCallum Cancer Centre, Parkville, Melbourne, Victoria Australia; 7 Sir Peter MacCallum Department of Oncology, The University of Melbourne, Parkville, Victoria Australia; 8 The Joint Pathology Center, Defense Health Agency, National Capital Region Medical Directorate, Silver Spring, MD USA; 9 Department of Anatomic Pathology, Division of Gynecologic Pathology, University of Southern California, Los Angeles, CA USA; 10 Center for Applied Proteomics and Molecular Medicine, George Mason University, Manassas, VA USA; 11 Center for Biomedical Informatics and Information Technology, National Cancer Institute, Rockville, MD USA; 12 Division of Cancer Epidemiology and Genetics National Cancer Institute, Rockville, MD USA; 13 Ellison Institute for Transformative Medicine, University of Southern California, Los Angeles, CA USA; 14 Department of Pathology and Laboratory Medicine, The University of British Columbia, Vancouver, British Columbia Canada; 15 Women’s Health Integrated Research Center, Women’s Service Line, Inova Health System, Falls Church, VA USA; 16 The Cancer Imaging Archive, National Cancer Institute, Bethesda, MD USA; 17 The Ohio State University, Columbus, OH USA; 18 Duke University Medical Center, Durham, NC USA; 19 University of Virginia, Charlottesville, VA USA; 20 University of Chicago, Chicago, IL USA; 21 MD Anderson Comprehensive Cancer Center, Houston, TX USA; 22 University of Cambridge, Cambridge, UK; 23 National Cancer Institute, Bethesda, MD USA; 24 Stanford University, Stanford, CA USA; 25 British Columbia Cancer Research Centre, Vancouver, Canada; 26 University of Oklahoma Health Sciences Center, Oklahoma City, OK USA; 27 The Australian Ovarian Cancer Study Group, Peter MacCallum Cancer Centre, Melbourne, Victoria Australia; 28 The Westmead Institute for Medical Research, Sydney, NSW Australia; 29 Department of Gynaecological Oncology, Westmead Hospital, Sydney, NSW Australia; 30 The University of Sydney, Sydney, NSW Australia; 31 Mayo Clinic, Rochester, MN USA

**Keywords:** Ovarian cancer, Oncology

## Abstract

We performed a deep proteogenomic analysis of bulk tumor and laser microdissection enriched tumor cell populations from high-grade serous ovarian cancer (HGSOC) tissue specimens spanning a broad spectrum of purity. We identified patients with longer progression-free survival had increased immune-related signatures and validated proteins correlating with tumor-infiltrating lymphocytes in 65 tumors from an independent cohort of HGSOC patients, as well as with overall survival in an additional 126 HGSOC patient cohort. We identified that homologous recombination deficient (HRD) tumors are enriched in pathways associated with metabolism and oxidative phosphorylation that we validated in independent patient cohorts. We further identified that polycomb complex protein BMI-1 is elevated in HR proficient (HRP) tumors, that elevated BMI-1 correlates with poor overall survival in HRP but not HRD HGSOC patients, and that HRP HGSOC cells are uniquely sensitive to BMI-1 inhibition.

## Introduction

Epithelial ovarian cancer is the fifth most common cause of cancer death among women in the US where 19,710 are predicted to be diagnosed with and 13,270 are predicted to succumb to ovarian cancer in 2023^1^. High-grade serous ovarian cancer (HGSOC) represents the most prevalent ovarian cancer histotype, where patients often present with advanced-stage disease and extensive disease burden. Although bevacizumab and poly [ADP-ribose] polymerase (PARP) inhibitors have provided exciting new treatment options for ovarian cancer patients, additional therapeutic options are needed for those with poor prognostic clinical features. This may in part be related to the diverse nature of HGSOC, which also has multiple prognostic molecular subtypes^2–5^. Recent investigations of these various molecular subtypes in HGSOC by our group^6^ and others^7^ have identified that the mesenchymal (MES) subtype is characterized by having a high proportion of stromal cells, correlating with low tumor purity. Historically, deep proteogenomic analyses of HGSOC have been conducted on bulk tumor tissues with inclusion criteria that biased the analysis towards high “purity” tumors (≥70% tumor cell nuclei)^8,9^. However, as many “impure” HGSOC tumors correlate with poor disease prognosis^3,4^, there exists the opportunity to add important new molecular knowledge by investigating HGSOC tumors across a broad purity continuum more reflective of the patient population.

To investigate proteogenomic alterations within the tumor epithelium in HGSOC, we employed laser microdissection (LMD) to enrich tumor cells in a cohort of 70 chemo-naive, advanced stage HGSOC patient tumors spanning a purity continuum of less than 20% to greater than 90% tumor cells. LMD enriched tumor (ET) collections underwent deep proteogenomic analysis including whole genome sequencing (WGS), transcriptomic, and multi-modal proteomic analyses, and were directly compared with parallel data levels generated from matched, bulk tumor (BT) tissue collections, along with a subset of cases from which the stromal compartment was enriched by LMD from the tumor microenvironment. A comprehensive and integrative analysis of these data identified and validated HGSOC tumor epithelial-specific proteogenomic alterations correlating with tumor purity, tumor-infiltrating lymphocytes (TILs), and disease prognosis, and identified an expression-based signature of homologous recombination deficiency (HRD).

## Results

### Ovarian cancer cohort characteristics and analyses

**Fig. 1:**
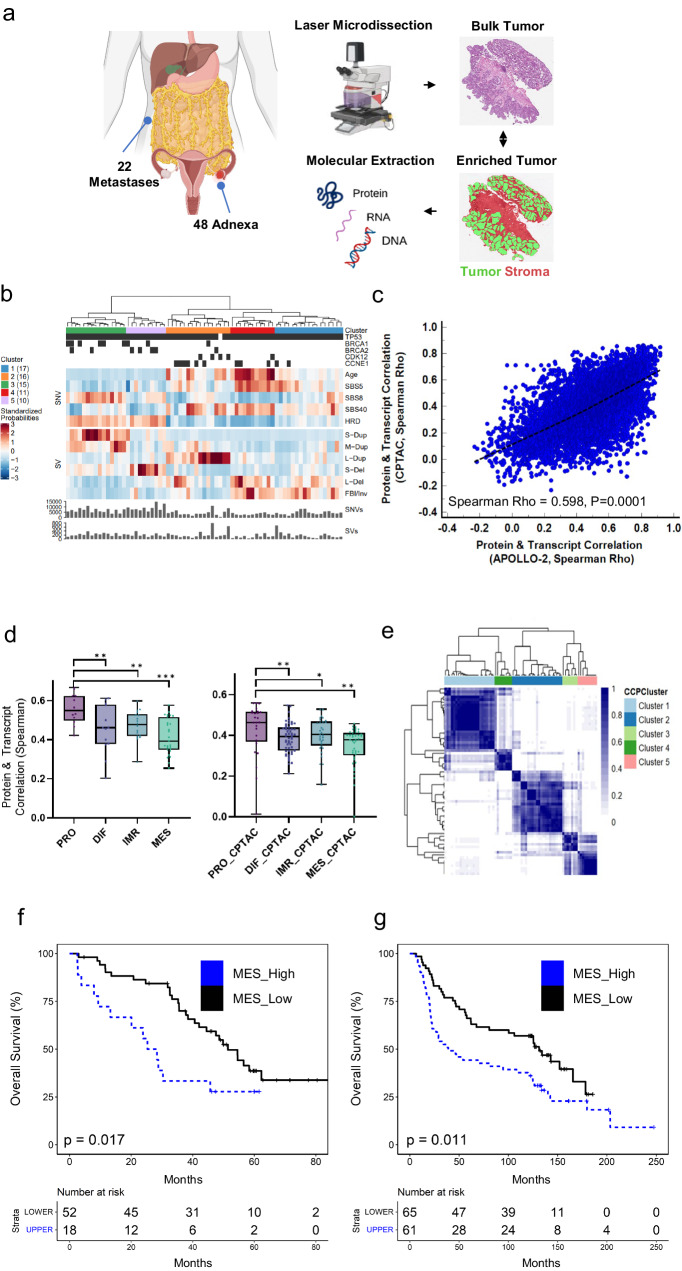
Proteogenomic analysis of high-grade serous ovarian cancer (HGSOC). **a** Bulk tumor (BT) and laser microdissection (LMD) enriched tumor (ET) epithelium from adnexal (*n* = 48) and metastatic (*n* = 22) HGSOC tumor specimens were prepared and DNA, RNA, and protein extracts were profiled by deep whole genome sequencing, RNA sequencing, and proteomics by mass spectrometry and reverse phase protein arrays; LMD was also used to collect stromal cell populations for a subset of cases (*n* = 48) for proteomics (Created in part with BioRender.com). **b** Hierarchical cluster analysis of somatic mutations (tumor protein 53, *TP53*), breast cancer type 1 and 2 susceptibility protein (*BRCA1/2*), cyclin-dependent kinase 12 (*CDK12*), copy number variation (cyclin E, *CCNE1*), SNV, and SV signatures previously investigated in HGSOC, and sum SNV and SV statistics per case in whole genome sequencing (WGS) data from BT collections. **c** Comparison of gene-wise protein and transcript correlations (5721 protein:transcript pairs) between BT data with HGSOC data from CPTAC HGSOC cohort (Spearman Rho = 0.598, *p* < 0.0001). **d** Comparison of protein and transcript correlations by molecular subtypes (ConsensusOV classifications, DIF – differentiated, IMR – immunoreactive, MES – mesenchymal, PRO – proliferative) classified using BT transcriptome data (*n* = 70) as well as for the CPTAC HGSOC cohort (*n* = 169); *designates *p*-values corresponding to Mann–Whitney U (MWU), *p* < 0.1, ** MWU *p* < 0.05, *** MWU *p* < 0.005. **e** Hierarchical analysis of consensus clusters calculated from the top 25% variably abundant proteins from 70 APOLLO-2 HGSOC BT proteome data. **f** Kaplan–Meier plot of overall survival in patients with tumors harboring high (MES_Upper: upper quartile, *n* = 18) versus low (MES_Lower: lower quartile, *n* = 52) correlation with nine prognostic proteins significantly elevated in MES vs DIF & PRO tumors (Log Rank, *p* = 0.017). **g** Kaplan–Meier overall survival curves from a 9-candidate expression signature correlating with differential tumor purity from an independent HGSOC cohort (*n* = 126)^15^ (Log-Rank, *p* = 0.011).

An integrative proteogenomic analysis was undertaken in bulk tumor (BT) collections and matched LMD procured tumor cells from 70 chemo-naive, HGSOC patient tumors (hereafter referred as the “APOLLO-2” cohort). Bulk tissue serial sections and matched LMD procured epithelial tumor cells were analyzed using four molecular profiling technologies: deep whole genome sequencing (WGS), mRNA sequencing (RNA-seq), mass spectrometry (MS)-based global proteomics, and reverse phase protein arrays (RPPA) (Fig. 1a, Table 1, Supplementary Data 1, and Supplementary Data 2). Our analytical strategy involved a comprehensive and integrative investigation of the proteogenomic data from BT tissue and LMD ET cells separately for each case as well as enriched stromal (ES) cells in a subset of cases.

**Table 1:** Clinical characteristics of the APOLLO-2 high grade serous ovarian cancer patient cohort

Clinical characteristic	Case (%)
*Age at diagnosis*	
<50 years old	5 (7)
50–59 years old	23 (33)
60–69 years old	27 (39)
70–80+years old	15 (21)
*Race and ethnicity*	
Non-Hispanic White	62 (89)
Non-Hispanic Black	4 (5.5)
Hispanic, Asian/Pacific Islander, Mixed	4 (5.5)
*Stage at diagnosis*	
III	52 (74)
IV	18 (26)
*Topographic site*	
Ovarian	36 (51)
Tubal	15 (21)
Tubo-Ovarian	13 (19)
Peritoneal	6 (9)
*Disease distribution*	
High	49 (70)
Moderate	17 (24)
Low	4 (6)
*Residual disease*	
R0 (none or microscopic)	27 (39)
R1 (macroscopic >1 cm)	43 (61)
*Progression-free survival*	
No Recurrence or Progression	10 (14)
Recurrence or Progression	60 (86)
*Vital status*	
Alive	26 (37)
Dead	44 (63)

### Molecular characterization of bulk tumor preparations

Patient tumors in the APOLLO-2 cohort had a median tumor purity of ~50%, admixed with varying levels of stromal and immune cells (Supplementary Data 2); tumor purity estimates calculated from WGS somatic mutation analysis correlated well with tumor purity assessments from expert pathology review (Spearman Rho = 0.561, *p* < 0.001, Supplementary Data 2). Single nucleotide variants (SNVs) and structural variant (SV) subtypes identified in the APOLLO-2 cohort were highly similar to those recently described for HGSOC^10^ (Fig. 1b, Supplementary Data). We identified common genomic signatures and relationships associated with HGSOC patient outcomes, including an association with superior outcome in patients (*n* = 18) with HRD-duplicated SV subtype tumors compared to those harboring fold-back inversions (FBI, *n* = 36, Log Rank, *p* = 0.0084, Supplementary Fig. 1A).

The overall protein:transcript pair (*n* = 7290) correlation quantified in global proteome and transcriptome analysis of BT collections for each patient tumor (Spearman Rho, R = 0.47) (Supplementary Data 2) was similar to BT samples from an independent proteogenomic analysis of HGSOC reported by the NCI’s Clinical Proteomics Tumor Assessment Consortium (CPTAC) (R = 0.47)^8^. We found a significant and large positive correlation of gene-wise transcript:protein correlation values between our APOLLO-2 cohort and the CPTAC HGSOC cohort (R = 0.598, *p* = 0.0001 from 5721 co-measured protein:transcript pairs, Fig. 1c). Protein:transcript correlation values were significantly associated with tumor purity (R = 0.44, *p* = 0.0001, Supplementary Data 2) and inversely correlated with immune (R = −0.223, *p* = 0.06) and fibroblast (R = −0.322, *p* = 0.007) scores calculated using Consensus^TME^^11^ gene signatures (Supplementary Fig. 1B). Assessment of consensus molecular subtypes from BT transcriptome data (ConsensusOV)^12^ showed that tumors classified as proliferative (PRO) had higher purity estimates and were comparable to differentiated (DIF) subtype tumors (~73.92% purity, *p* = 0.8623, Supplementary Fig. 1C, Supplementary Data 2). Tumors classified as immunoreactive (IMR) or MES had significantly lower WGS tumor purity estimates than PRO tumors (IMR vs PRO, *p* = 0.035 and MES vs PRO, *p* = 0.0004, respectively; Supplementary Fig. 1C). Evaluation of transcriptome-derived immune scores calculated using Consensus^TME^ as a surrogate of immune cell admixture or fibroblast scores as a surrogate of stromal cell admixture demonstrated that IMR tumors had higher immune scores (*p* = 0.014) and MES tumors had higher fibroblast scores (*p* < 0.0001) compared to other tumor types (Supplementary Data 2). We identified significantly higher protein:transcript correlation values in PRO tumors (average R = 0.55) than DIF (R = 0.46, *p* = 0.02), IMR (R = 0.48, *p* = 0.0089), or MES (R = 0.39, *p* = 0.0003) tumor subtypes in the APOLLO-2 cohort, a finding we validated in data from the CPTAC HGSOC cohort (Fig. 1d).

Integration of weighted gene co-expression network analysis (WGCNA)^13^ with hierarchical cluster analysis of BT proteome data identified five primary clusters (Fig. 1e, Supplementary Data 2). These clusters align with conventional HGSOC prognostic molecular subtypes and are strongly correlated with pathways enriched in WGCNA modules (Supplementary Fig. 1D)^12^. Comparison of these results with similar WGCNA analysis of proteomic data from bulk HGSOC tumor proteomic data from CPTAC^8^ showed a high conservation of Hallmark pathways and protein alterations within modules identified between these two independent cohorts prepared and analyzed as bulk tumor tissue (Supplementary Fig. 1E, Supplementary Data 3).

As MES tumors have been correlated with high stromal cell admixture and worse disease prognosis^3,6^, we were motivated to investigate protein alterations correlating with tumor purity and patient prognosis. We identified that a high proportion of cluster 1 tumors (Fig. 1e) classified as the MES subtype are from metastatic loci (Fisher’s Exact, *p* = 0.0001, Supplementary Data 2). Recently, Eckert et al.^14^ identified a protein signature of cellular stroma in adnexal and omental metastasis in HGSOC tumors. Correlation of the 47 stromal signature proteins from Eckert et al. co-quantified in enriched stroma collections from our APOLLO-2 cohort (*n* = 32 adnexal and *n* = 16 metastatic specimens) showed that metastatic stromal proteins were highly correlated with omental metastasis (Mann–Whitney U, MWU, *p* = 0.0025; Supplementary Fig. 1F). A differential analysis of BT proteome data from low purity MES tumors (*n* = 27) with high purity DIF (*n* = 13) and PRO (*n* = 12) tumors identified 653 significantly altered proteins (LIMMA, adjusted *p* < 0.05), among which nine proteins were significantly correlated with overall survival (OS, multivariate Wald *p* < 0.05 adjusting for patient age, disease stage and residual disease status, Supplementary Data 4). Each of these nine proteins were correlated with an increased risk of death and all, except for intraflagellar transport 122 (IFT122) and dynein cytoplasmic 2 light intermediate chain 1 (DYNC2LI1), were elevated in MES tumors. Patients whose tumors were highly correlated with the abundance of these nine prognostic proteins (upper quartile, *n* = 18), experienced significantly shorter OS (Log Rank, *p* = 0.017) compared to the rest of the cohort (lower quartiles, *n* = 52, Fig. 1f). We further identified the relationship of these features with poor disease outcome remained significant following multivariate analysis as noted above further adjusting for treatment with neoadjuvant chemotherapy, PARP inhibitor or mutational status for *BRCA1* or *2* (aHR = 2.23, 1.07–4.65, *p* = 0.032, Supplementary Data 5). We investigated this association at the transcript level in an independent HGSOC cohort that includes a population of exceptional survivors^15^ and found that patients with a high (*n* = 61 tumors) vs. low (*n* = 65 tumors) correlation have an increased risk of death (Log Rank, *p* = 0.011, Fig. 1g, Supplementary Data 5). We evaluated a subset of these proteins mapping to data from a recent study of intratumoral proteogenomic heterogeneity in HGSOC tumors conducted by our group^6^ and identified that most of these proteins are significantly elevated in stroma relative to tumor cells (MWU, *p* < 0.05) (Supplementary Fig. 1G). We also evaluated transcript level data derived from bulk tissue collections for an independent cohort of 129 patient tumors recently reported^15^ relative to estimates of tumor purity and identified that the abundance of these nine transcripts are significantly, inversely correlated with tumor purity (R = −0.381, *p* < 1E−4). Among these prognostic proteins, metalloproteinase inhibitor 3 (TIMP3, continuous Wald, *p* < 0.05 and Log-Rank *p* < 0.05, Supplementary Fig. 1H) and matrix remodeling-associated protein 8 (MXRA8, continuous Wald, *p* < 0.05) are also significantly associated with OS in proteomic data from the CPTAC HGSOC cohort.

### Molecular characterization of enriched tumor preparations

**Fig. 2:**
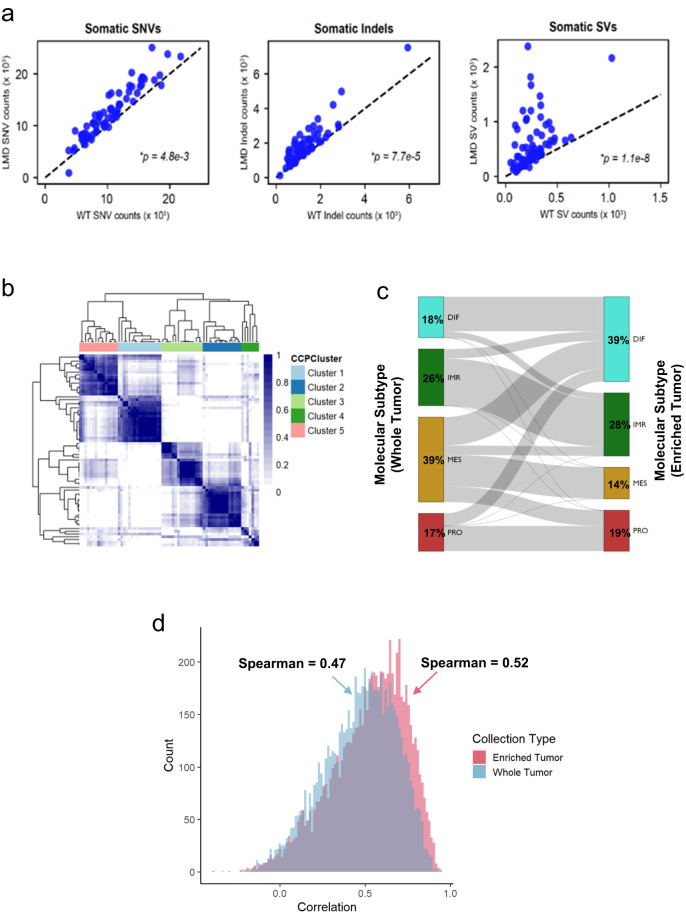
Proteogenomic analysis of high-grade serous ovarian cancers (HGSOC) - characterization of enriched tumor collections. **a** Single nucleotide variants (SNVs), insertion/deletion (indel), and structural variants (SVs) identified from whole genome sequencing (WGS) data from laser microdissection (LMD) enriched tumor (ET) cell populations compared to bulk tumor (BT) tissue collections from the same specimen (*p reflects significance of Spearman Rho). **b** Hierarchical analysis of consensus clusters calculated from top 25% most variably abundant proteins from HGSOC ET proteome data (*n* = 70). **c** Sankey plot illustrating the transition of molecular subtypes classified by ConsensusOV in BT and ET transcriptome data. **d** Correlation analysis of global proteome and transcriptome data (7209 protein:transcript pairs) in BT collections (Spearman Rho = 0.47) is significantly lower (Mann–Whitney U, MWU *p* = 0.0007) than the correlation (Spearman Rho = 0.52) in ET collections (7598 protein:transcript pairs).

In addition to proteogenomic analysis of bulk tissue in the APOLLO-2 HGSOC cohort, LMD was used to selectively harvest tumor epithelium from serial histologic sections for each of the 70 HGSOC cases followed by comprehensive molecular analyses (WGS, RNA-seq, MS-proteomics, and RPPA). As anticipated, we found that LMD enrichment significantly increased the median tumor purity as estimated by WGS as compared to BT tissue (83.5% vs 62.5%, respectively, MWU *p* < 0.0001, Supplementary Data 2). Analysis of WGS from ET samples showed substantial and significant increases in the identification of somatic single nucleotide variants (SNV, MWU *p* = 4.8e^−3^), indel *(p* = 7.7e^−5^), and structural variants (SV, *p* = 1.1e^−8^) as compared to matched BT tissue (Fig. 2a). Although we did not identify new recurrent somatic gene mutations or SNV or SV subtype classifications between ET and BT WGS data, we did identify significant increases in variant allele frequencies of somatic mutations in ET WGS data (e.g., *TP53*, Supplementary Fig. 2A), along with significant increases in predicted neoepitopes observed in both WGS as well as RNA-seq data (Supplementary Fig. 2B, Supplementary Data 2).

Evaluation of the ET WGS data showed that IMR and MES tumors have significantly lower purities than DIF tumors (Supplementary Fig. 1C, 67.6%, *p* = 0.005 and 72.9%, *p* = 0.003, respectively) and have higher immune cell and fibroblast scores, respectively (*p* ≤ 0.005, Supplementary Data 2), whereas DIF and PRO tumors have comparable tumor purities (~84.5%, *p* = 0.2761). We conducted global MS-based proteomic analyses of BT and ET collections for a subset of metastatic tumors from ten patients from whom we also analyzed BT and ET proteomes from the matched adnexal tumor specimen (Supplementary Data 2). This paired analysis demonstrated that BT proteomic profiles generally cluster by anatomic location whereas ET proteomic profiles cluster in a patient centric manner, suggesting that molecular profiles in ET preparations are highly conserved irrespective of whether the tumor is from the adnexal or metastatic location (Supplementary Fig. 2C, comparing average distances between matched tumors for BT vs ET collections, *p* = 0.002).

An unsupervised analysis of ET proteome data identified 5 predominant consensus clusters that are associated with conventional HGSOC molecular subtypes; cluster 1 is significantly associated with PRO, cluster 2 with DIF, cluster 3 with IMR, and cluster 5 with MES tumors (Fisher’s Exact, *p* < 0.05, Fig. 2b), and are enriched for similar pathways (Supplementary Fig. 2D). Cluster 4 is comprised predominantly of IMR and DIF tumors and a differential analysis compared to other clusters identified 145 significantly altered proteins (LIMMA, *p* < 0.01, Supplementary Data 6) that, from GSEA, are associated with pathways regulating mitosis, cell cycle, and cytoskeletal organization.

Comparison of molecular subtypes in BT and ET collections showed that a significant number of tumors classified as MES (*n* = 17, 39%) from BT transcriptome data transition to DIF (*n* = 9) as well as IMR (*n* = 3) or PRO (*n* = 5) when classified from ET transcriptome data (Mann–Whitney U *p* = 0.0016, Fig. 2c). This analysis is consistent with a recent study published by our group^6^ showing that molecular subtype classifications are impacted by tumor purity. To this end, we performed a correlation analysis of molecular subtype classifications for tumor cores, enriched and bulk tumor collections for a single HGSOC patient tumor (Fig. 5a)^6^, and identified molecular subtypes are well correlated between tumor cores and enriched tumor collections (Spearman Rho = 0.81 ± 0.21), but poorly correlated between tumor cores and whole tumor collections (average Spearman Rho = 0.161 ± 0.71, MWU *p* < 1E−4, Supplementary Fig. 3), owing largely to differential tumor purity. Pathology review of tumors that did not reclassify from MES following LMD enrichment showed that the tumor epithelial cells were highly infiltrated with stromal fibroblast cell populations that were not effectively decoupled using LMD. Analysis of gene-wise protein:transcript abundance correlation values for each patient tumor showed a significantly lower median correlation for BT (R = 0.47) compared to ET collections (R = 0.52, MWU, *p* = 0.0007, Fig. 2d). We investigated protein:transcript abundance correlation for tumors exhibiting the largest difference in WGS-informed tumor purity between BT and ET collections; the purity of these 23 tumors increased by an average of 38.7% ± 7.7% (lower tertile, Supplementary Data 2), and found that the median correlation for BT collections was significantly lower (R = 0.379) versus enriched tumor collections (R = 0.496, MWU, *p* = 0.0002) for these tumors. In summary, comparison of overarching pathways enriched following hierarchical cluster analysis shows that most tumors are explained by conventional HGSOC molecular subtypes and tumor purity, with lower purity MES tumors having the greatest propensity to be reclassified to other molecular subtypes due to the enrichment of tumor epithelium from stromal cells by LMD.

### Identification of expression patterns that correlate with immune cell infiltration and disease prognosis

**Fig. 3:**
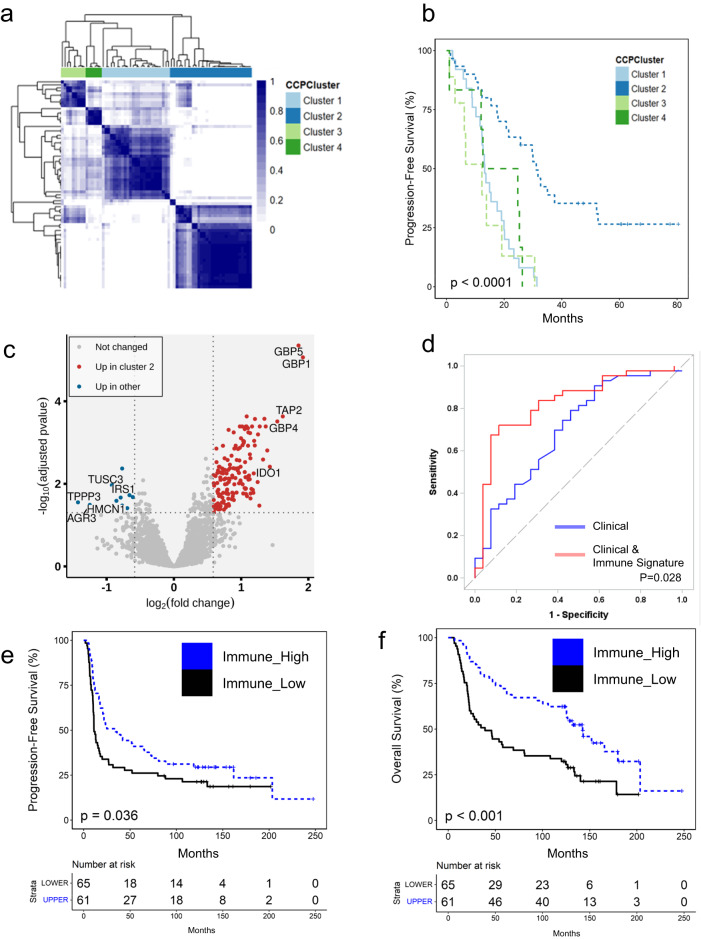
Molecular alterations associated with immune cell infiltration, cell heterogeneity, and disease prognosis. **a** Hierarchical cluster analysis of 69 proteins and 257 transcripts correlating with progression-free survival (PFS) identified by univariate (continuous Cox and log-rank, *p* < 0.05) and multivariate analysis, adjusting for patient age, disease stage, and residual disease status (continuous Cox Chi-Square, *p* < 0.05). **b** Kaplan–Meier plot of PFS from the four primary patient consensus clusters described in panel (**a**). **c** Differential analysis of enriched tumor proteomes in consensus cluster plus (CCP) 2 vs CCP1/3/4 identified 166 significantly altered proteins (LIMMA, adjusted *p* < 0.05, ±1.5 fold-change). **d** Classification of disease recurrence risk based on clinical variables, i.e., patient age, disease stage, residual disease status, *BRCA1/2* mutation status, and PARP-inhibitor treatment alone (blue line, baseline AUC = 0.701) or in conjunction with exhibiting high correlation with 15 candidate immune signature (AUC = 0.829, *P* = 0.028). **e** Kaplan–Meier PFS curves from an analysis of HGSOC long-term survivor tumors with high (*n* = 61 tumor samples) vs. low (*n* = 65 tumor samples) correlation with a 15 candidate expression signature of immune cell infiltration (Log Rank, *p* = 0.036) **f** Kaplan–Meier overall survival curves from an analysis of HGSOC long-term survivors with high (*n* = 61) vs. low (*n* = 65) correlation with a 15 candidate expression signature of immune cell infiltration (Log Rank, *p* < 0.001).

Using both univariate (continuous Cox and categorized Log-Rank, *p* < 0.05) and multivariate analyses as described above (continuous Chi-Square, *p* < 0.05), we identified 69 proteins and 257 transcripts from ET datasets associated with progression-free survival (PFS, Supplementary Data 7). One candidate, NEK9 (NIMA Related Kinase 9), was significantly correlated with altered disease prognosis at both the transcript and protein level. Hierarchical analysis of these candidates shows that patient tumors organize into four consensus clusters (Fig. 3a). We also identified that patients in cluster 2 (*n* = 30) have a significantly longer progression-free interval (~1.5 years) than patients in the other clusters (Fig. 3b, Log-Rank, *p* < 0.0001) and a significantly lower risk of death (Log Rank, *p* = 0.001, Supplementary Fig. 4A, Supplementary Data 8). We further identify cluster 2 patients experience improved disease prognosis following multivariate analysis (aHR, for progression-free interval = 0.17, 0.09–0.35, Wald *p*-value < 1E−4 and for overall survival aHR = 0.32 (0.15–0.68), Wald *p*-value = 0.003). We also assessed whether cluster 2 patients were likely to be enriched for somatic copy number variations (CNVs), CCNE1 amplification, tumors classified as HRD by CHORD score, or to have mutations in *BRCA1*, *BRCA2* or other DNA damage response (DDR) genes recently described by Garsed et al.^15^ Our results showed cluster 2 patients are more likely to harbor mutations in DDR genes compared to other patients in our cohort (odds ratio, OR = 3.43, 95% CI = 1.24–9.44, Fisher’s Exact, *p* = 0.02). DDR genes implicated included *ATM, ATR, BRCA1, BRCA2, CDK12, CHEK1, CHEK2, FANCM,* and *RB1* (Supplementary Data 2). A high proportion of cluster 2 patient tumors were classified as IMR (Fisher’s Exact, *p* = 0.0001), had higher immune scores (MWU, *p* < 0.003), and were more likely to have immune cells present as determined by expert pathology review (Fisher’s Exact *p* = 0.022, Supplementary Data 2). Using CIBERSORTX^16^, we found that cluster 2 patient tumors were enriched with M1 macrophages, CD8 T-cells, and plasma cells (LIMMA, *p* < 0.05, Supplementary Data 9). Differential analysis of ET proteomic data identified several significantly elevated proteins in cluster 2 that are associated with immune cell activation, including interferon-induced guanylate-binding protein 1 and 5 (GBP1 and GBP5)^17^, cluster of differentiation 38 (CD38)^18^, and antigen processing including transporter associated with antigen processing 1 and 2 (TAP1/2)^19^ (LIMMA, adjusted *p* < 0.05, Fig. 3c, Supplementary Data 10). Data from RPPA showed that cluster 2 patients had significant (LIMMA, adjusted *p* < 0.05) elevations in leukocyte common antigen, CD45^20^, major histocompatibility complex subunit, β_2_ macroglobulin^21^, as well as growth factor receptor-bound protein 2 (GRB2), the latter of which plays important roles in immune cell regulation^22^ (Supplementary Data 11). Despite these signs of anti-tumor immunity, there were no significant differences in the proportion or binding affinities of neoepitopes predicted in cluster 2 versus other patient tumors (Supplementary Data 2, data not shown). We further investigated the literature for drivers of immune exclusion and identified the discoidin domain receptor 1 (DDR1) as regulating this event within other solid tumor malignancies^23^. We then compared DDR1 protein abundance relative to immune scores (ConsensusTME) using bulk and enriched tumor collections and identified DDR1 protein as inversely correlated with immune scores in bulk (Rho = −0.372, *p* = 0.0015), and trending as such in enriched tumor (Rho = −0.18, *p* = 0.136) collections.

A recent analysis of the HGSOC tumor microenvironment described by Zhang et al. illustrated that TILs have discrete infiltration patterns into both tumor epithelium and stroma (ES-TIL type) or are restricted to stroma (S-TIL); it was also identified that ES-TIL and S-TIL patients have a better disease prognosis in comparison with patient tumors with low or no immune cell infiltration (N-TIL)^24^. To further explore this immune infiltration pattern at the protein level, we procured representative specimens from the Zhang et al. cohort and conducted a quantitative MS-based proteomic analysis of ES-TIL (*n* = 11 tumor samples), S-TIL (*n* = 12), and N-TIL (*n* = 42) BT tissues (Supplementary Data 12). Consistent with transcript-level evidence from Zhang et al., our proteome-level data similarly suggested that ES-TIL and S-TIL tumors have significantly higher immune scores than N-TIL tumors (Supplementary Fig. 4B). The proteome profile of ES-TIL tumors strongly correlated with cluster 2 tumors, but not with N-TIL tumors (MWU, *p* = 0.0024) (Supplementary Fig. 4C). We identified 15 proteins significantly altered between cluster 2 versus other patient tumors that were strongly associated with PFS (multivariate Chi-Square, *p* < 0.05). We further investigated the impact of high correlation with these 15 features on prediction of disease recurrence along with clinical variables known to correlate with this risk (Fig. 3d) and identified that integration of all features was correlated with a significant improvement in predicting disease recurrence (AUC = 0.829) in comparison with clinical variables alone (AUC = 0.701, *p* = 0.028). These 15 proteins were also significantly associated with ES-TIL (median R = 0.48) (Supplementary Fig. 4D) and IMR tumors in the CPTAC HGSOC cohort (MWU, *p* < 0.0001, Supplementary Fig. 4E)^8^. We further investigated protein abundance of DDR1 in the cohort from Zhang et al. and identified this protein is significantly decreased in ES & S-TIL in comparison to N-TIL tumors (ES & S-TIL vs N-TIL: −0.42 logFC, LIMMA p = 0.02). We investigated the relationship of these 15 TIL-related proteins in transcript-level data from a cohort of 126 HGSOC patients characterized by long-term (>10 years, *n* = 60), moderate-term (3–9 years, *n* = 32) and short-term (<2 years, *n* = 34) OS^15^ and found significant associations with PFS (Log Rank, *p* = 0.036, Fig. 3e) and OS (Log Rank, *p* < 0.001, Fig. 3f, Supplementary Data 8). We investigated this TIL-related protein panel in the CPTAC HGSOC cohort and, although none of these proteins were independently associated with PFS or OS, patients with tumors having higher expression correlation values (R ≥ 0.5, *n* = 17) for these fifteen proteins had an 80% lower risk of death (odds ratio, OR = 0.2, 95% CI = 0.055–0.72, Fisher’s Exact, *p* = 0.014) in comparison to patients with tumors with lower correlations (R ≤ −0.5, *n* = 31). This finding is strongly supported by transcript level evidence from TCGA where HGSOC patients with tumors that correlated with this feature set (upper quintile, *n* = 97) were less likely to die compared to lower quartiles (*n* = 392, OR = 0.58, 95% CI = 0.37–0.91, Fisher’s Exact, *p* = 0.02, Supplementary Fig. 4F).

### Identification of HRD-associated proteins and transcripts in enriched tumor cell populations

**Fig. 4:**
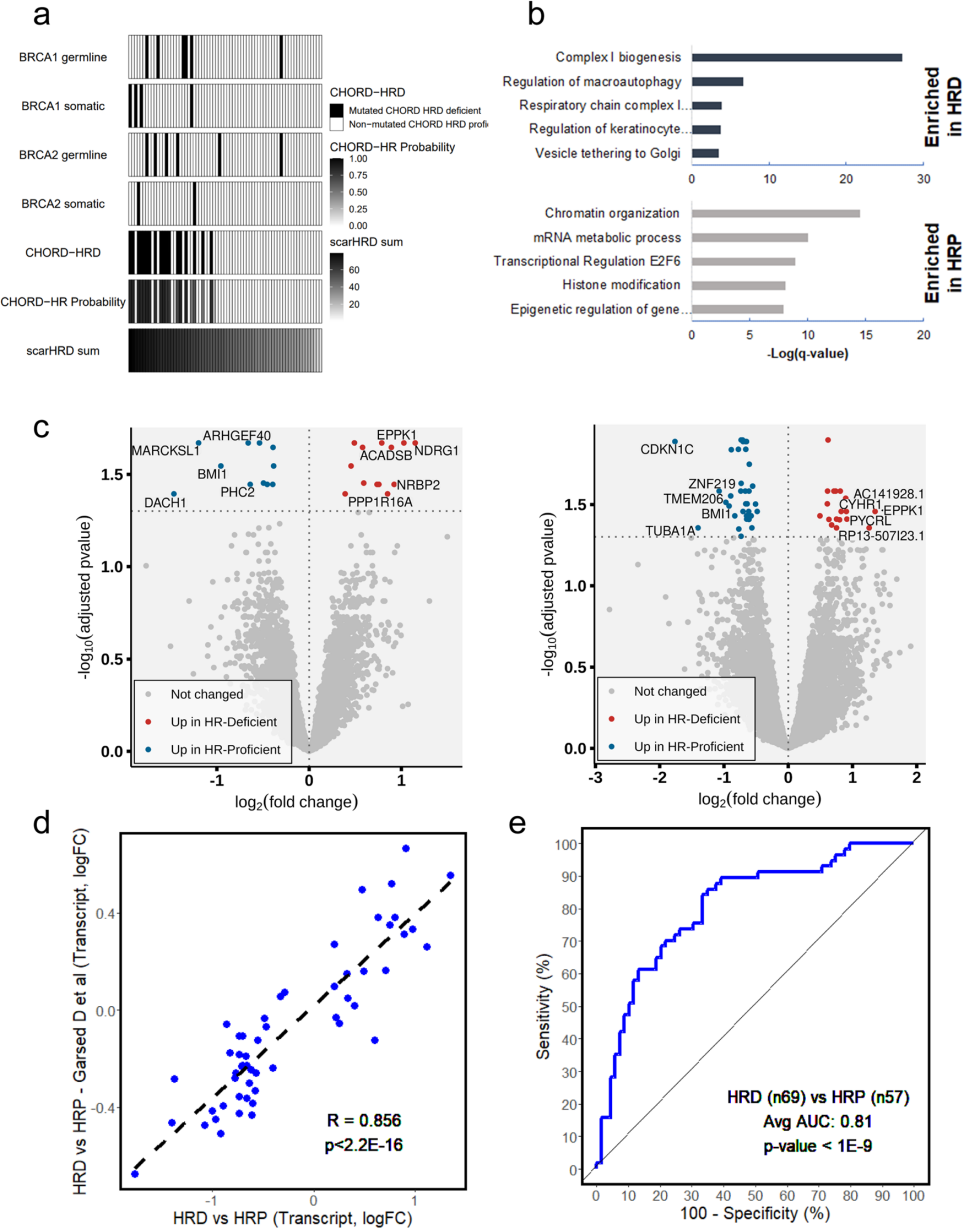
Identification of proteins and transcripts in enriched tumor cell populations associated with homologous recombination deficiency in high-grade serous ovarian cancer. **a** Integration of germline and somatic mutation status for breast cancer type 1 and 2 susceptibility protein (*BRCA1/2*), and classification of tumors as homologous recombination deficient (HRD) or proficient (HRP) by probability of HRD (continuous) by CHORD score and scarHRD. **b** Top pathways enriched among proteins significantly altered (LIMMA *p*-value < 0.01) between patient tumors classified as HRD or HRP; default settings in metascape.org. **c** Differential analysis of enriched tumor cell proteome (left panel) and transcript (right panel) data from HRD (*n* = 18) vs HRP (*n* = 51) patients (LIMMA, adjusted *p* < 0.05). **d** Correlation analysis of 54 HRD-associated transcripts with an independent cohort of HGSOC patients classified as HRD (*n* = 69) or HRP (*n* = 57) by CHORD score (R = Spearman Rho). **e** Classification of HRD (*n* = 69) vs HRP (*n* = 57) tumors using an integrated 54 protein/transcript HRD expression signature in an independent cohort of HGSOC patients (receiver operating characteristic curve, AUC = 0.81, *p* < 1E−9).

Homologous recombination deficiency status was determined from BT WGS-derived somatic mutation data by estimating telomeric allelic imbalance, loss of heterozygosity, and the number of large-scale transitions using scarHRD^25^, as well by a random forest classifier developed from a pan-cancer analysis of HRD tumors (CHORD score)^26^. Eighteen tumors were classified as HRD using CHORD that also have significantly higher scarHRD scores than HR proficient (HRP) classified tumors (MWU, *p* < 0.0001, Supplementary Data 2), many of which not surprisingly harbor germline or somatic alterations in *BRCA1* and *BRCA2* genes (Fig. 4a), genetic alterations known to underpin HRD^26^. Patient tumors classified as HRD for which we did not identify *BRCA1* or *BRCA2* mutations did have lower levels of the *BRCA1* transcript relative to HRP tumors and we identified that the *BRCA1* gene promoter was significantly (LIMMA, adjusted *p* < 0.05) hypermethylated in these cases compared to others (Supplementary Data 2, Supplementary Data 13). Patients with tumors classified as HRD by CHORD had a significantly lower risk of death relative to patients with HRP tumors (OR = 0.31, 95% CI = 0.1–0.94, Fisher’s Exact, *p* = 0.039). We did not, however, observe significantly different immune scores (ConsensusTME) between tumors classified as HRD (average score = 0.01) versus HRP (average score = −0.008, MWU, *p* = 0.31).

A differential analysis of HRD vs HRP from ET specimen MS-proteomics data identified 350 significantly altered proteins (LIMMA, *p* < 0.01), many of which are involved in pathways regulating mitochondrial and metabolic activity in HRD tumors (Supplementary Data 14). Of note, we observed a marked elevation of core subunits of mitochondrial complex I (Fig. 4b, Supplementary Data 14) in HRD tumors, which we found to not likely be from altered mitochondrial load based on an orthogonal immunohistochemical analysis of COX-IV^27^ in a subset of HRD (*n* = 8) and HRP (*n* = 9) patient tumors (Supplementary Data 2). We compared protein alterations in matched metastases for two HRD patients (A072, A096) and identified 92 proteins elevated in these tumors compared to metastatic specimens from HRP patients, and these again were associated with pathways regulating mitochondrial regulation and metabolic activity. We also compared protein alterations between HRD (*n* = 13) and HRP (*n* = 35) tumors for a cross-section of our cohort with global proteome data generated for matched enriched tumor and stroma collections (Supplementary Fig. 5). We identified little overlap of significantly altered proteins between enriched tumor or enriched stroma populations between HRD and HRP tumors (Supplementary Fig. 5A) and observed enrichment of pathways regulating mitochondrion organization in tumor, but not stroma cell populations (Supplementary Fig. 5B). We further compared proteins significantly altered between HRD and HRP tumors with differences in tumor purity estimates between bulk and enriched tumor collections and identified that high correlation of protein alterations in bulk and enriched tumor collections was negatively correlated with tumor purity differences (Spearman Rho = −0.562, *p* = 0.046, Supplementary Fig. 5C). These analyses suggest the HRD associated expression features prioritized are highly specific for tumor cell populations.

We next sought to identify whether proteins and/or transcripts identified in HRD versus HRP tumors could effectively classify tumors based on HR status. A differential analysis of proteome and transcriptome level data identified 54 altered protein and transcript candidates between HRD and HRP tumors (LIMMA, adjusted *p* < 0.05, Fig. 4c, Supplementary Data 15). Further investigation identified five of these candidates are significantly co-altered (LIMMA adjusted p < 0.05) at protein and transcript levels and exhibit concordant abundance trends, including EPPK1 and Pyrroline-5-carboxylate reductase (PYCRL) elevated in HRD vs HRP tumors and BMI1 Proto-Oncogene, Polycomb Ring Finger (BMI1), WD Repeat Domain 41 (WDR41), KH RNA Binding Domain Containing, Signal Transduction Associated 1 (KHDRBS1) reduced in HRD vs HRP tumors. There are also 36 candidates co-quantified at the protein and transcript levels with significantly correlated abundance trends in HRD vs HRP tumors (Spearman Rho = 0.813, *p* < 1E−4). Using sparse Partial Least Squares Discriminant Analysis (sPLS-DA), we found that this expression-based signature could classify HR status (*n* = 18 HRD and *n* = 51 HRP) with high sensitivity and specificity based on ET transcript data (RNA-seq, average AUC = 0.987 following 1000 iterations in sPLS-DA, average *p* < 1.01E−9). We assessed performance of this signature in transcript-level data (RNA-seq) in an independent HGSOC cohort (*n* = 69 HRD and n = 57 HRP) from Garsed et al. where HR was also classified from WGS data by CHORD^15^. Not only did we identify a strong quantitative correlation with the Garsed et al. cohort (Fig. 4d, R = 0.856, *p* < 2.2E−16), but further identified that the APOLLO-2 54 transcript signature classified HR status in the Garsed et al. cohort with high sensitivity and specificity (Fig. 4e, average AUC = 0.81 for 1000 iterations in sPLS-DA considering coefficients identified in our training analysis noted above in our model, average *p* < 3.35E−9). Finally, we evaluated the performance of our HRD expression signature in proteome- and transcriptome-level data from CPTAC and TCGA, respectively^8,9^. We found that 33 candidates from our HRD signature mapped to global proteome data from the CPTAC HGSOC cohort^8^ and identified that these are strongly correlated with *BRCA1* and *BRCA2* mutant (*n* = 15) compared to wild type (*n* = 125) CPTAC HGSOC tumors (Supplementary Fig. 6A, R = 0.493, *p* = 0.004). In TCGA, 43 of the 54 transcripts mapped to microarray gene expression data and we also found these to be highly correlated between *BRCA1* and *BRCA2* mutant (*n* = 67) or wild type (*n* = 422) HGSOC tumors (R = 0.74, *p* < 1E−6, Supplementary Fig. 6B)^9^.

### Pharmacologic small molecule BMI1 inhibitors selectively kill HRP HGSOC cells

**Fig. 5:**
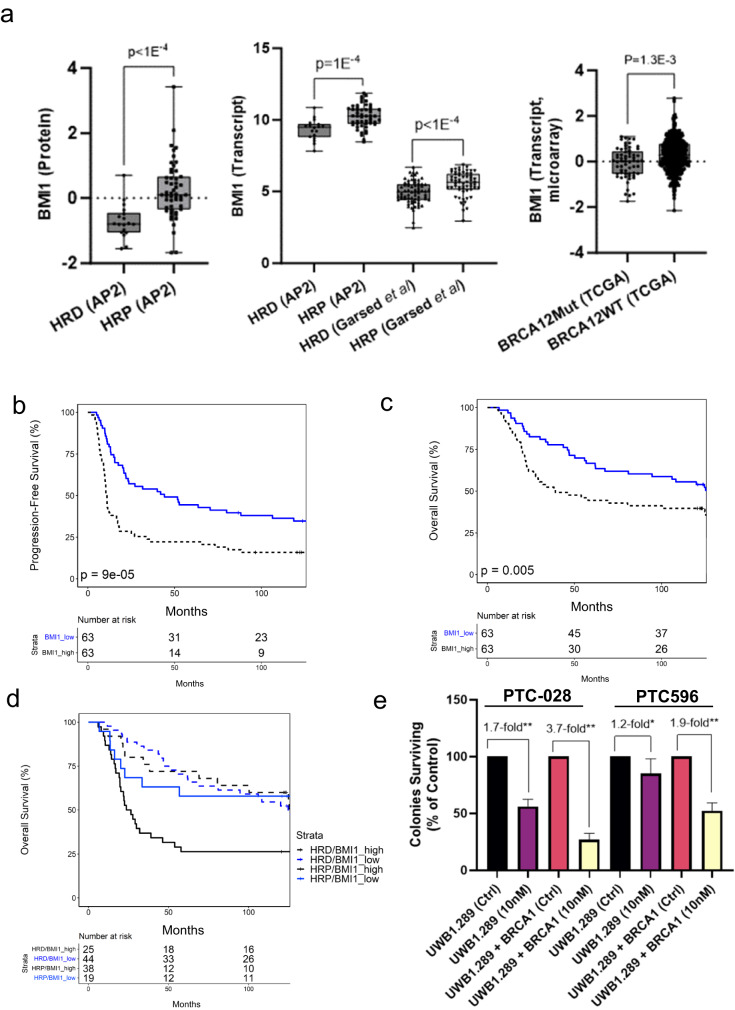
Polycomb complex protein BMI-1 is elevated in homologous recombination proficient (HRP) HGSOC tumors and HRP HGSOC cells exhibit increased sensitivity to pharmacologic BMI1 inhibitors. **a** BMI1 protein abundance in HRD (*n* = 18, APOLLO-2, AP2) and HRP (*n* = 51, AP2) and BMI1 transcript abundance in HRD (AP2 & *n* = 69, multidisciplinary ovarian cancer outcomes group cohort, MOCOG) and HRP (AP2 & *n* = 57, MOCOG) as well as in HGSOC tumors with (*n* = 61, TCGA) or without (*n* = 391, TCGA) mutations in *BRCA1* or *BRCA2*; *p*-value reflects Mann–Whitney U rank sum testing. **b** Progression-free survival curves for HGSOC patients with BMI1 high (BMI1_high, *n* = 63) versus low (BMI1_low, *n* = 63) BMI1 transcript levels; *p*-value reflects Log Rank testing. **c** Overall survival curves for HGSOC patients with BMI high (BMI1_high, *n* = 63) versus low (BMI1_low, *n* = 63) BMI1 transcript levels; *p*-value reflects Log Rank testing. **d** Overall survival curves for HGSOC patients with BMI high (BMI1_high) versus low (BMI1_low) BMI1 transcript levels stratified by HRD (*n* = 69, multivariate *p*-value = 0.153, log rank *p*-value = 0.44) and HRP (*n* = 57, multivariate *p*-value = 0.02, log-rank *p*-value = 0.026) status (Supplementary Data 16). **e** Results from colony survival assays for UWB1.289 and UWB1.289 + BRCA1 treated with BMI1 inhibitors PTC-028 or PTC-596; *p*-value reflects Mann–Whitney U rank sum testing (**p = 0.0021, *p = 0.04); the results reflect three independent, biological replicate experiments.

Arising from the lack of therapeutic options for HRP HGSOC patients and the absence of “targetable” driver mutations in this population, we sought to uncover putative drug targets in our expression level data. We identified that BMI1 is significantly elevated at the protein and transcript level in HRP HGSOC tumors^26^ in our APOLLO-2 cohort, as well as in the Garsed et al.^15^ cohort and in HGSOC tumors without mutations in *BRCA1* or *BRCA2* from the TCGA^9^ study (Fig. 5a). We also find that elevated BMI1 expression is correlated with an increased risk of disease progression (aHR = 2.39, Chi-square *p* = 7E−5, Fig. 5b) in a cohort of 126 HGSOC patients^15^ and worse overall survival in this same cohort (aHR = 2.12, *p* = 1E−3, Supplementary Fig. 5C) and an independent cohort of 440 HGSOC patients^9^ (aHR = 1.34, *p* = 0.016, Supplementary Fig. 7A) following multivariate analysis (aHR for Garsed et al. reflects 122 patients, excluding 4 patients that received neoadjuvant chemotherapy, Supplementary Data 16). We also identified that elevated BMI1 is correlated with worse disease outcome in HRP (*n* = 57, aHR = 2.47, *p* = 0.02), but not HRD (*n* = 69, aHR = 1.6, *p* = 0.153, Fig. 5d) HGSOC patients, as well as in HGSOC patients with wild-type *BRCA1* or *BRCA2* (*n* = 379, aHR = 1.36, *p* = 0.02) compared to patients harboring mutations in these genes (*n* = 61, aHR = 1.00, *p* = 0.997, Supplementary Fig. 7B) following adjustment for covariates noted above (Supplementary Data 16). We investigated the impact of BMI1 inhibition in a previously described^28^ isogenic cell line model of HRD (UWB1.289) and HRP (UWB1.289 + BRCA1) HGSOC cells. We confirmed BRCA1 expression and assessed BMI1 protein levels in UWB1.289 + BRCA1 versus UWB cells (Supplementary Fig. 7C). We then assessed two pharmacologic small molecule inhibitors of BMI1 (PTC-028^29^ and PTC596) in these cell lines by colony survival assay and observed that UWB1.289 + BRCA1 cells are >2-fold and >1.5-fold more sensitive to PTC-028 and PTC596, respectively (Fig. 5e, Supplementary Data 17).

## Discussion

Our study employed LMD to conduct an integrated proteogenomic analysis of matched, BT and ET collections from 70 HGSOC tumors. Key findings from our comparison of BT and ET show that most MES tumors classified from BT collections reclassify to the DIF subtype in ET collections. Recent evidence from our group^6^ and others^7,30^ has shown that MES tumors are typified by having high stromal cell content and the data presented here corroborates these findings at a cohort level. Our data show that the protein:transcript correlation values are higher in high purity PRO subtypes versus the lower purity IMR and MES subtypes. Recent evidence from our group^6^ and others^31,32^ has shown that protein:transcript abundance correlations are lower in normal tissues in comparison to tumor cells. Our finding that molecular subtypes with high proportions of normal cell populations (e.g., immune and stromal cells) also have lower protein:transcript correlation values are consistent with these previous reports. Our data demonstrate significant increases in sensitivity for identifying somatic mutations and structural variants from WGS generated from ET samples and, most notably, higher proportions of predicted neoepitopes. This latter finding suggests that LMD enrichment of tumor epithelium may improve the coverage of neoepitopes and further that this workflow may better support personalized immunotherapy workflows, such as adoptive T-cell transfer.

We identified nine proteins elevated in MES tumors that correlate with an increased risk of death and validated this association in an independent cohort of HGSOC patients (*n* = 126), which includes a large proportion of exceptionally long-term survivors ( > 10 years)^15^. Of note, we found that most of these proteins are elevated in stromal compared to tumor cells and inversely correlated with tumor purity. We validated a number of these proteins in CPTAC HGSOC data, namely TIMP3, a protease localized to the extracellular matrix that has previously been correlated with poor OS in HGSOC^33^, and MXRA8, a transmembrane protein that has been identified as a marker of cancer-associated fibroblasts in pancreatic cancer^34^ that also correlates with poor outcome in glioma^35^.

Unsupervised analysis of the proteomic data from ET collections resulted in a cluster of HGSOC patients characterized by a significantly longer progression-free interval (~1.5 years) than the other patients in our cohort. We identified that these patient tumors have transcript signatures consistent with the presence of immune cells. As enrichment of immune transcript signatures within LMD-ET collections suggests intratumoral immune cell infiltration, we were motivated to correlate proteome alterations identified within our cohort with an independent cohort of tumor specimens derived from BT proteomics data from three major TIL HGSOC subtypes: ES-TIL (tumors with substantial levels of both epithelial and stromal TILs) S-TIL (tumors dominated by stromal TILs), and N-TIL (tumors sparsely infiltrated by TILs). We found that proteins associated with longer PFS were most highly correlated with ES-TIL tumors, followed by S-TIL, and less so in those classified as N-TIL. We identified fifteen proteins that strongly correlated with PFS in our cohort, independent of common covariates of disease progression (e.g., patient age at diagnosis, disease stage and residual disease status), that included several proteins known to regulate antigen presentation^36^ (TAP2, adjusted hazard ratio, aHR = 0.76, *p* = 0.02), T-cell activation^37^ (SPN, aHR = 0.7, *p* = 0.014) and have further been correlated with regulating immune cell infiltration in other organ-site malignancies^38^ (EMC2, aHR = 0.27, *p* = 4.4E−4). We investigated these immune-related candidates in transcript data from an independent cohort of 126 HGSOC patients^15^, many of whom survived greater than 10 years, and confirmed that these 15 immune-related proteins strongly correlate with PFS and OS. We further identified the discoidin domain receptor 1 (DDR1), a protein kinase shown to promote immune exclusion in other solid tumor malignancies^23,39^, as inversely proportional to immune scores calculated using ConsensusTME from companion transcriptome data, suggesting similar roles for this protein in HGSOC. We found that this immune-related protein signature was associated with a lower risk of death in HGSOC patients in CPTAC^8^ and TCGA^9^ data. Hence, this analysis identified and validated proteins correlating with immune cell infiltration and improved disease prognosis, and future efforts will be focused on investigating the role of these candidates in regulating immune surveillance of HGSOC tumor cells.

We explored expression alterations in LMD ET cell populations from HRD or HRP tumors and identified proteins associated with pathways regulating mitochondrial and metabolic activity uniquely elevated in HRD tumor epithelium. Recent evidence has shown that HR deficient breast and ovarian cancers have an increased dependency on oxidative phosphorylation (OXPHOS) rather than glycolysis for energy metabolism and that HRD tumor cells have elevated complex I respiratory chain subunits, such as NADH:Ubiquinone Oxidoreductase Core Subunit V2 (NDUFV2)^40–42^. We found several mitochondrial complex I subunits to be elevated in HRD tumors, which we demonstrated by IHC was not likely due to differential mitochondrial load within cellular subpopulations. We further compared proteins altered in enriched tumor and stroma populations from HRD and HRP tumors and confirmed the alterations correlating with altered metabolism and mitochondria likely originate from tumor, not stroma cell populations. We identified a combined protein and transcript signature that enabled the classification of HR status with high predictive accuracy, which we validated in an independent cohort of HGSOC tumors^15^. Additional investigation of our HR expression signature in independent CPTAC^8^ and TCGA^9^ HGSOC tumor cohort data showed a high quantitative correlation to *BRCA1* or *BRCA2* mutational status. These analyses identified and validated a non-gene centric, yet highly accurate, expression signature for classifying HRD status in HGSOC tumors. Future efforts will be focused on exploration of expression alterations we have identified as altered between HRD and HRP tumors with the goal of defining mechanistic contributions to the HRD phenotype.

Our proteomics analysis identified that HRP tumors are enriched in pathways regulating chromatin and DNA replication, including elevated polycomb complex protein BMI-1 (HRP vs HRD logFC: +0.96, adjusted *p* = 0.03), which is a protein involved in regulating homologous recombination repair^43^ and is the target of oral, small molecular BMI-1 inhibitors^44^. We further identified elevated transcript and protein levels of BMI1 in both HRP and *BRCA1/2* wild-type HGSOC tumors. That BMI1 is involved in regulating homologous recombination repair^43^ and is the target of pharmacologic small molecule inhibitors (PTC028^29^ and PTC596^44^), we sought to further understand whether this protein represented a hitherto unrecognized player in the ovarian cancer HRP phenotype. Notably, we find that elevated BMI1 correlates with worse overall survival following multivariate analysis in >500 HGSOC patients between two independent cohorts^9,15^ and, furthermore, observe that this relationship remains significant in tumors classified as HRP or as having wild-type *BRCA1* or *BRCA2* in comparison with HRD tumors or as having mutated *BRCA1/2*. We further identify that HRP (UWB1.289 + BRCA1) HGSOC cells exhibit increased sensitivity to PTC-028 and PTC596 than HRD (UWB1.289) HGSOC cells. Notably, we find that UWB1.289 + BRCA1 cells are more sensitive to PTC-028, a drug that has been shown to induce cellular apoptosis via degradation of BMI1^29^, than PTC-596, which has also been shown to degrade BMI1 but to additionally inhibit tubulin polymerization resulting in apoptosis^45^. The elevated abundance of BMI1 in HRP HGSOC tumor cells and the recently described^43^ role of BMI1 in regulating homologous recombination repair supports the increased sensitivity of HRP (UWB1.289 + BRCA1) cells to PTC-028 and suggests these cells are more dependent on BMI1 than HRD (UWB1.289) HGSOC cells. Additional investigation of BMI-1 abundance and overall survival in the Garsed et al. cohort identified that there is no significant difference between disease outcome for HRP/BMI1 low tumors in comparison to HRD tumors regardless of BMI1 abundance status, suggesting that inhibition of BMI1 in HRP backgrounds could in concept phenocopy HRD through promotion of an HR deficient phenotype. This further suggests that combination treatment with BMI1i and a poly (ADP-ribose) polymerase inhibitor may represent a novel targeted therapeutic strategy for HRP HGSOC patients and investigating this combination as well as performing further confirmatory investigations of BMI1 inhibitor sensitivity in HRP HGSOC cell line backgrounds will be the focus of future efforts. These results, paired with the unique relationship of BMI1 abundance with disease outcome in HRP, but not HRD patients, suggest that targeting BMI1 may represent a hitherto unrecognized therapeutic opportunity in HRP proficient HGSOC tumors.

The clinical applications of these data are innovative and particularly applicable to ovarian cancer patients with poor prognostic features. Approximately 70% of the patients in this cohort had disease in the upper abdomen and despite aggressive cytoreductive procedures, ~60% of patients had visible residual at the time of primary debulking surgery. Inclusion of patients with widespread disease distribution patterns led to analysis of tumors across a wide spectrum of tumor purity (10–90%, mean 50%) unlike TCGA and CPTAC which included tumors with >70% tumor cell nuclei. Although the number of patients characterized as HRD is lower than the generalized population of ovarian cancer patients^46^, this reflects the aggressive cancer phenotypes included in our cohort. Using enrichment techniques, we have demonstrated significant increases in sensitivity for identifying somatic mutations and structural variants from WGS generated from ET samples (compared to BT preparations) and, most notably, higher proportions of predicted neoepitopes. This latter finding suggests that LMD enrichment of tumor epithelium may improve the coverage of neoepitopes, and this finding suggests that this workflow may better support personalized immunotherapy workflows, such as adoptive T-cell transfer. The prognostic relevance of our immune and the purity-associated expression signatures remained significantly predictive of disease outcomes following multivariate modeling with prediction models being validated in independent case sets. Our data verify that historical expression-based tumor types are largely reflective of tumor purity and that multiple prognostically relevant proteins are actually stromal in origin. Our data have further identified multiple examples of targetable candidates identified through enrichment techniques that would otherwise have been missed with analysis of BT. In summary, our proteogenomic analysis provides important new clinically relevant insights into HGSOC tumor cell populations, and have uncovered prognostic proteogenomic alterations correlating with TILs, low tumor purity, as well as expression alterations associated with HRD status and immune infiltration.

## Methods

### Patient cohort

Fresh-frozen tumor tissues and blood samples were selected from patients enrolled in the WCG IRB approved (#20110222) Tissue and Data Acquisition Study of Gynecologic Disease who underwent primary debulking surgery or a diagnostic laparoscopy at Inova Fairfax Medical Campus (Inova), Duke University Medical Center (Duke) or the Ohio State University (OSU); all experimental protocols involving human data in this study were in accordance with the Declaration of Helsinki and written informed consent was obtained from all subjects involved in the study. Patients receiving neoadjuvant chemotherapy prior to surgery were not eligible for analysis. Most tumor tissues were collected from adnexal sites (*n* = 49), such as the ovary (*n* = 48) or fallopian tube (*n* = 1) with the remainder being collected from metastatic sites (*n* = 21), such as the omentum (*n* = 12) or other organ sites (*n* = 9) (Supplementary Data 2). Representative hematoxylin and eosin-stained tissue sections generated for all tumor samples underwent expert pathology review by a board-certified pathologist (BAC and/or PMF). Pathology review confirmed a diagnosis of high grade serous ovarian cancer and provided relative proportions of cellular subpopulations of interest.

### Tissue collections and molecular extraction for proteogenomic analysis

Fresh-frozen patient tumors were embedded in optimal cutting temperature (OCT) compound and sectioned (8 µm) onto glass slides for hematoxylin and eosin (H&E) staining for pathology review or onto polyethylene naphthalate (PEN) membrane slides for laser microdissection. All PEN membrane sections were stained with H&E and stains for sections destined for LMD harvests for nucleic acid extraction included RNase inhibitors (RNAProtect, Sigma Aldrich). Tissue sections destined for DNA, RNA, and protein extractions were generated from sequential sections generated from each patient tumor block. Laser microdissection was performed (LMD 6500, Leica Microsystems, Wetzlar, Germany) to harvest cellular populations of interest from pathologically-defined regions. Enrichment of tumor and stroma cell populations was performed to achieve greater than 95% purity for each cellular population, avoiding regions of fat or necrosis.

### Preparation of DNA samples

Germline DNA was extracted from patient blood samples (*n* = 69) and tumor DNA was collected from tumor scrolls (BT collections) or by LMD (ET cell populations) (*n* = 69). Samples were collected directly into microfuge tubes supplemented with ATL buffer (Qiagen Sciences, LLC, Germantown, MD). Samples were normalized to 360 µL ATL buffer and 40 µL of proteinase K was added for lysis and incubated at 56 °C for 4 h with intermittent shaking. DNA isolation was performed according to the manufacturer’s protocol (DNA Purification from Tissues) using the QiAamp DNA Mini Kit (Qiagen Sciences, LLC). DNA was eluted after a 10 min incubation with 40 µL of Buffer AE, followed by another 10 min incubation with 160 µL of nuclease-free water (Thermo Fisher Scientific) and reduced to 50 µL by vacuum centrifugation (CentriVap Concentrator, Labconco, Kansas City, MO). Quantity and purity (260/280 ratio) were assessed spectrophotometrically (Nanodrop 2000 Spectrophotometer, Thermo Fisher Scientific, Inc.) and fluorometrically (Quant-iT PicoGreen dsDNA Assay Kit, Thermo Fisher Scientific) according to manufacturer’s protocols.

### Preparation of RNA samples

Tissue sections on PEN membrane slides were manually scraped (BT collections, representing all tissue sectioned onto a slide) or underwent LMD (ET collections, representing ET cell populations) directly into Buffer RLT with β-mercaptoethanol (Qiagen). RNA was purified using the RNeasy Micro Kit (Qiagen) per the Purification of Total RNA from Microdissected Cryosections Protocol including on-column DNase digestion. RNA concentrations were determined using Qubit RNA High Sensitivity kit (Thermo Fisher Scientific, Inc.). RNA integrity numbers (RIN) were calculated using the RNA 6000 Pico Kit on the 2100 Bioanalyzer (Agilent Technologies, Santa Clara, CA).

### Specimen preparation for mass spectrometry-based proteomics and reverse phase protein arrays

Collection of HGSOC cancer tissues using LMD, sample digestion, preparation of TMT multiplexes and offline, basic reversed-phase liquid chromatographic (bRPLC) fractionation was performed essentially as previously described^47,48^. Briefly, BT, ET, and ES were harvested by LMD; the average tissue area collected per sample was 70 mm^2^ (BT and ET, *n* = 70) or 25 mm^2^ (enriched stroma, *n* = 48). Enriched tumor samples for reverse phase protein array (RPPA) analysis were collected using LMD as described above into SDS lysis buffer. Bulk tumor collections from fresh-frozen tissues for HGSOC tumors previously described by Zhang et al.^24^ were also collected for quantitative proteomic analysis as described below. Samples were collected into 20 µL of 100 mM TEAB/10% acetonitrile, pH 8.0 in MicroTubes (Pressure BioSciences, Inc, South Easton, MA) and were lysed and digested with a heat-stable form of trypsin (SMART Trypsin, Thermo Fisher Scientific, Inc.) employing pressure cycling technology with a barocycler (2320EXT Pressure BioSciences, Inc). Peptide digests were transferred to 0.5 mL microcentrifuge tubes, vacuum dried, resuspended in 100 mM TEAB, pH 8.0 and peptide concentration was determined using the bicinchoninic acid assay (Thermo Fisher Scientific, Inc.). Equivalent amounts of peptide (40 µg for ET and BT and 5 or 10 µg for enriched stroma), along with a reference sample generated by pooling equivalent amounts of peptide digests from individual patient samples, were aliquoted into a final volume of 100 µL of 100 mM TEAB and labeled with tandem-mass tag (TMT) isobaric labels (TMT-11plex™ Isobaric Label Reagent Set, Thermo Fisher Scientific, Inc.) according to manufacturer’s recommendations. Each TMT-11 multiplex was loaded onto a C-18 reversed-phase liquid chromatography trap column in 10 mM NH_4_HCO_3_ (pH 8.0) and resolved into 96 fractions through development of a linear gradient of acetonitrile (0.69% acetonitrile/min) on a 1260 Infinity II liquid chromatograph (Agilent Technologies). For ET and BT TMT multiplexes, concatenated fractions (36 pooled samples representing 10% of the entire peptide sample) were generated for global LC-MS/MS analysis. For ES, 36 concatenated fractions were generated using 100% of the samples for global LC-MS/MS analysis.

### DNA PCR-free library preparation and whole genome sequencing

TruSeq DNA PCR-free Library Preparation Kit (Illumina, San Diego, CA) was performed following manufacturer’s instructions. Briefly, genomic DNA (gDNA) was diluted to 20 ng/μL using Resuspension Buffer (RSB, Illumina) and 55 μL was transferred to Covaris microTubes (Covaris, Woburn, MA). The normalized gDNA was then sheared on an LE220 focused-ultrasonication system (Covaris) to achieve target peaks of 450 bp with an Average Power of 81.0 W (SonoLab settings: duty factor, 18.0%; peak incident power, 45.0 watts; 200 cycles per burst; treatment duration, 60 s; water bath temperature, 5–8.5 °C). The quality of the final DNA libraries was assessed (High Sensitivity dsDNA, AATI) as per manufacturer’s protocol; library peak size was in the range of 550 to 620 bp. The DNA libraries were quantified by real-time quantitative PCR, using the KAPA SYBR FAST Library Quantification Kit (KAPA Biosystems, Boston, MA) optimized for the Roche LightCycler 480 instrument (Roche, Indianapolis, IN). Low input amount samples were libraried using the Illumina DNA PCR-free Prep, Tagmentation and IDT for Illumina DNA/UD Indexes Set A (Illumina, CA) with minor modifications to the manufacturer’s protocol for automation and incubation on a Hybex incubator. Single stranded sequencing libraries were not assessed for size distribution. DNA libraries were then normalized to 2 nM and clustered on the Illumina cBot 2 at 200 pM using a HiSeq X Flowcell v2 and the HiSeq X HD Paired-End Cluster Generation Kit v2. Paired-end sequencing was performed with the HiSeq X HD SBS Kit (300 cycles) on the Illumina HiSeq X. Tagmentation-based sequencing libraries were sequenced on a NovaSeq 6000 (Illumina, CA) using a NovaSeq S4 Flowcell and SBS Kit (300 cycles). Mean genome coverage was >30X for germline DNA samples and >90X for tumor DNA samples. WGS sample raw reads were aligned to the hg38 reference genome and further processed through the Resequencing workflow within Illumina’s HiSeq Analysis Software (HAS; Isis version 2.5.55.1311; https://support.illumina.com/sequencing/sequencing_software/hiseq-analysis-software-v2-1.html). This workflow utilizes the Isaac read aligner (iSAAC-SAAC00776.15.01.27) and variant caller (starka-2.1.4.2)^49^, the Manta structural variant caller (version 0.23.1)^50^, and the Canvas CNV caller (version 1.1.0.5)^51^. Tumor purity estimates were derived tumor WGS data by Canvas in the Illumina Tumor Normal Workflow^52^.

### RNA-seq analyses and data processing

Sequencing libraries were prepared from 500 ng of total RNA input using the TruSeq Stranded mRNA Library Preparation Kit (Illumina) with index barcoded adapters. Sequencing library yield and concentration were determined using the Illumina/Universal Library Quantification Kit (KAPA Biosystems) on the CFX 384 real time system (Bio-Rad, Hercules, CA). Library size distribution was determined using the Fragment Analyzer TM (Advanced Analytical Technologies, Inc, Ames, IA) with adapter dimer contamination confirmed to be less than 0.3%. Clustering and sequencing were performed on the HiSeq 500 (Illumina) using a High Output 150 cycle kit for paired-end reads of 75 bp length and an intended depth of 50 million reads per sample. RNA sequencing data were aligned to HG38 and processed to normalized gene expression values as previously described^52^. Raw mapped read counts underwent VST normalization using DESeq2 (3.14).

### Liquid chromatography-tandem mass spectrometry

Liquid chromatography-tandem mass spectrometry (LC-MS/MS) analyses of TMT-11 multiplexes was performed essentially as previously described^47^. In brief, each concatenated TMT fraction (5 μL, ~600 ng) was loaded on a nanoflow high-performance LC system (EASY-nLC 1200, Thermo Fisher Scientific) employing a two-column system comprised of a reversed-phase trap column (Acclaim^TM^ PepMap^TM^ 100, 75 μm × 2 cm, nanoViper, Thermo Fisher Scientific) and a heated (50 °C) reversed-phase analytical column (PepMap^TM^ RSLC C18, 2 μm, 100 Å, 75 μm × 50 cm, nanoViper, Thermo Fisher Scientific) connected online with an Orbitrap mass spectrometer (Q Exactive HF-X, Thermo Fisher Scientific). Peptides were eluted by developing a linear gradient of 2% mobile phase A (2% acetonitrile, 0.1% formic acid) to 32% mobile phase B (95% acetonitrile, 0.1% formic acid) within 120 min at a constant flow rate of 250 nL/min. High-resolution (R = 60,000 at *m/z* 200) broadband (*m/z* 400–1600) mass spectra (MS) were acquired, from which the top 12 most intense molecular ions in each MS scan were selected for high-energy collisional dissociation (HCD, normalized collision energy of 34%) acquisition in the Orbitrap at high resolution (R = 60,000 at *m/z* 200). Spray voltage was set to 2.1 kV, S-Lens RF level was set to 40%, and capillary temperature was set to 275 °C. Peptide molecular ions selected for HCD were restricted to z = 2–4 and both MS1 and MS2 spectra were collected in profile mode. Dynamic exclusion (*t* = 20 s at a mass tolerance = 10 ppm) was enabled to minimize redundant selection of peptide molecular ions for HCD. Mass spectrometry data files were searched against a publicly available, non-redundant human proteome database (Swiss-Prot, Homo sapiens, http://www.uniprot.org) using Mascot (Matrix Science, Boston, MA, USA), Proteome Discoverer (Thermo Fisher Scientific) and in-house tools using identical parameters as previously described^47,48^. Reproducibility of LC-MS/MS analysis was further monitored by assessing peptide spectral match (PSM) identifications following analyses of a commercial human cell line digest (PRV6951, Fisher Scientific) before and after analysis of each TMT patient sample multiplex. These results demonstrated exceptionally stable analytical performance over the course of LC-MS/MS analysis of the APOLLO-2 cohort (4.8% CV) (Supplementary Data 18).

### Reverse phase protein array

Tissue lysates derived from LMD were kept at −80 °C until they were immobilized onto nitrocellulose coated slides (Grace Bio-labs, Bend, OR) using an Aushon 2470 arrayer (Aushon BioSystems, Billerica, MA); case A044 was excluded from RPPA analysis due to insufficient material. Each sample was printed in technical triplicates along with reference standards used for internal quality control/assurance. To estimate the amount of protein in each sample, selected arrays (one in every 15) were stained with Sypro Ruby Protein Blot Stain (Molecular Probes, Eugene, OR) following manufacturer’s instructions^53,54^. Prior to antibody staining, the arrays were treated with Reblot Antibody Stripping solution (Chemicon, Temecula, CA) for 15 min at ambient temperature, washed with PBS and incubated for 4 h in I-block (Tropix, Bedford, MA). Arrays were then probed with 3% hydrogen peroxide, a biotin blocking system (Dako Cytomation, Carpinteria, CA), and an additional serum free protein block (Dako Cytomation) using an automated system (Dako Cytomation) as previously descried^54^. Each array was then probed with one antibody targeting an unmodified or a post-translationally modified epitope. Antibodies were validated as previously described^55^. Slides were then probed with a biotinylated secondary antibody matching the species of the primary antibody (anti-rabbit and anti-human, Vector Laboratories, Inc. Burlingame, CA; anti-mouse, CSA; Dako Cytomation). A commercially available tyramide-based avidin/biotin amplification kit (CSA; Dako Cytomation) coupled with the IRDye680RD Streptavidin fluorescent dye (LI-COR Biosciences, Lincoln, NE) was employed to amplify the detection of the signal. Slides were scanned on a laser scanner (TECAN, Mönnedorf, Switzerland) using the 620 nm and 580 nm wavelength channels for antibodies and total protein slides, respectively. Images were analyzed with a commercially available software (MicroVigene 5.1.0.0; Vigenetech, Carlisle, MA) as previously described^53^; this software performs automatic spot finding and subtraction of the local background along with the non-specific binding generated by the secondary antibody. Finally, each sample was normalized to its corresponding amount of protein derived from the Sypro Ruby stained slides and technical replicates were averaged. RPPA antibody identifiers were mapped to UniProt protein accessions and HGNC identifiers through manual inspection of commercial antibody names and corresponding human protein entries curated within the UniProt resource. Pan-specific antibodies were assigned to multiple protein isoform accessions and residues for modified proteins were mapped to curated protein model positions.

### Immunohistochemical analysis of cytochrome c oxidase subunit 4 (COX-IV)

Immunohistochemistry (IHC) was performed on fresh-frozen tissue sections from representative tumors in our APOLLO-2 cohort classified as HRD (*n* = 8) or HRP (*n* = 9) by CHORD score. Slides were fixed with 100% methanol, 5 min at ambient temperature followed with a PBS rinse. Ambient temperature incubations in 0.5% triton-PBS for 15 min and 2.5% normal goat serum, 30 min, were used to permeabilize the tissue and block nonspecific protein binding. The slides were incubated overnight at 4 °C with anti-COX IV antibody - Mitochondrial Loading Control, rabbit polyclonal (Abcam, Waltham, MA, ab16056, 1:1000). Dako’s Envision diaminobenzidine (DAB) detection system was used to label and color bound protein complexes. Normal lung tissue was used as the positive tissue control. Following detection, slides were counterstained with hematoxylin then dehydrated and coverslipped. Stained tissue sections were scanned on an Aperio ScanScope XT slide scanner (Leica Microsystems).scanner and digital images underwent expert pathology review (PMF).

### DNA extraction, methylation array, and data processing

DNA purified from tissue samples described above was analyzed at the Cancer Genomics Research Laboratory in the Division of Cancer Epidemiology and Genetics at the National Cancer Institute. Briefly, DNA concentration was determined by the Quant-iT PicoGreen dsDNA assay (ThermoFisher Scientific) and 400 ng was treated with sodium bisulfite using the EZ-96 DNA Methylation MagPrep Kit (Zymo Research, Irvine, CA) according to manufacturer’s protocol. Bisulfite-treated samples were denatured and neutralized, then whole genome amplified isothermally, to increase the amount of DNA template. Methylation was measured using the Infinium MethylationEPIC BeadChip (Illumina Inc.), which interrogates over 850,000 CpG sites in the genome. Samples were run in a single batch. DNA extracted from a laboratory internal control cell line, NA07057 (Coriell Cell Repositories, Camden, NJ), was utilized to confirm the efficiency of bisulfite conversion. In addition, three samples were run in duplicate, and correlations of methylation values for these duplicates were greater than 0.99. All samples passed internal quality control.

Methylation array raw data files (idat files) were processed with the minfi R package. Methylation values with detection *p* values > 0.01 were assigned as missing, as these are intended to identify low quality probes by comparing methylation signal at each probe from negative controls probes. Probes with >25% missing values were removed (*n* = 1027). Other excluded probes were those that were cross-reactive (*n* = 43,079)^56^ and those in the Y chromosome (*n* = 70). A total of 822,682 CpGs were included in the analysis. Missing methylation values were imputed with the R function impute.knn (k = 5). Beta values were normalized using the BMIQ method. The ComBat function was used to adjust the methylation values for batch effects. CpG sites were considered to be in the promoter region if they were located within 200 bp or 1500 bp upstream of the transcription start site (TSS), 5′ UTRs, or exon 1, based on the manifest file of the Illumina MethylationEPIC array^57^. Analysis of methylation probes mapping to *BRCA1* were prioritized for downstream analysis.

### Bioinformatics analyses

Sample matching of BT and ET collections was confirmed by head-to-head comparison of orthogonal data levels generated for each sample including (1) comparison of WGS and RNA-seq data by hierarchical clustering of pairwise genotype distances among select single nucleotide variants (SNV) in germline WGS, BT and ET WGS and RNA-seq was performed as sample co-clustering were considered matched and (2) correlation analysis of RNA-seq and MS-based proteomics data for BT and ET collections were compared separately where the rank abundance of co-quantified proteins and transcripts were correlated and samples with the highest correlation (Spearman Rho) with cognate transcriptome and proteome data derived from the sample patient tumor samples were considered matched. Differential analyses of global proteome or transcriptome matrixes were performed using the LIMMA package (version 3.8) in R (version 3.5.2). Pathway analysis was performed using Metascape (https://metascape.org/gp/index.html#/main/step) using default parameters. Molecular subtype analysis was generated by consensusOV (version 1.12.0) from whole and enriched transcript matrices and transitions were plotted as a Sankey plot with networkD3 (version 0.4) in R Studio (version 3.6.0). The clinical outcomes included progression-free survival (PFS) and overall survival (OS). PFS was defined as the time from diagnosis until disease progression or death from any cause, whichever occurred first. OS was defined as the time from diagnosis until death from any cause. Associations with PFS and OS were evaluated using Cox modeling and Kaplan–Meier methods. For Kaplan–Meier analyses, high versus low expression and correlation with signature candidates of interest was defined by the median cut-point. Multivariate analysis was performed with adjustments for age (continuous variable), disease stage (III vs. IV), and residual disease status (residual vs no residual) for those biomarkers with univariate *p* values < 0.05. Kaplan–Meier analyses of prognostic signatures significantly correlating with altered disease prognosis further underwent multivariate analyses adjusting for the clinical variables noted above as well as treatment with neoadjuvant chemotherapy, i.e., NACT (tumor collected during diagnostic surgery prior to NACT), or PARP inhibitors during adjuvant or maintenance treatment as well as for *BRCA1/2* mutation status using SASSurvival analyses were conducted using the survival package (version 2.37-7) in R (version 3.12) and SAS (version 9.4). Global proteomics data for the CPTAC ovarian cancer cohort was downloaded from the data supplement in Zhang et al.^8^ and proteins quantified in >50% of patient samples were imputed using identical parameters as previously described^47,48^. Microarray gene expression data from the TCGA ovarian^9^ cancer cohort was downloaded from cbioportal.org. RNA-seq data described by Garsed et al. was provided by collaborators at the Peter MacCallum Cancer Centre^15^, and proteomic data from Hunt et al.^6^ was analyzed at lmdomics.org.

### Consensus cluster plus

The top 25 percent most variably abundant (mean absolute deviation, MAD) proteins from the BT and ET MS-proteomic analyses were included in the cluster analysis. The proteome matrices were protein-wise median centered as per the ConsensusClusterPlus documentation. For the proteogenomic ConsensusClusterPlus PFS signature, the enriched transcriptome matrix was first subsetted to significant transcript PFS signature candidates prior to z-score normalization. It was then combined with the proteome enriched matrix, subset to proteome-specific significant PFS candidates, and the entire matrix is median centered per the ConsensusClusterPlus documentation. The following parameters were used in the ConsensusClusterPlus (version 1.48.0) algorithm: seed 378, 1000 iterations, hierarchical clustering algorithm with distance calculated by pearson correlation, and defaults for all other parameters. Final cluster designations were selected on criteria previously described^58^. Clusters comprised of fewer than five samples were reassigned to the larger clusters previously assigned to maximize cohort size.

### sPLS-DA analysis

The sparse partial least squares discriminant analysis (sPLS-DA) model was first optimized on a 70:30 percent split of the transcript data to optimize the number of components selected for the final model (mixOmics version 6.8.5; caret version 6.0-86). Transcript data was subset to the final significant candidate panel list, 54 genes and proteins that overlap with an independent validation dataset and pass an adjusted *p*-value < 0.05, prior to running the sPLS-DA model on 2 components. Performance of the model on the training dataset was modeled by the average area under the receiver operating curve (AUROC) by averaging the HRD and HRP predicted distances over 1000 iterations. Performance was assessed in the testing dataset by predicting classification on the Mahalanobis distance and generating a ROC curve (pROC version 1.16.2).

### Weighted correlation network analysis

The co-expression network was constructed through the “WGCNA” package (version 1.69) in the R environment (version 3.6.2), the top 25% MAD proteins from BT and the top 25% MAD proteins from ET data, derived from the ConsensusClusterPlus analysis. The WGCNA settings were with soft thresholding power = 9, minimum module size = 25, medium sensitivity (deepSplit) = 2, and the signed method were used to group the genes into modules. Based on the hierarchical clustering and gene set analysis of modules, smaller modules adjacent in the cluster tree and modules with similar biological functions were merged. The correlation heatmap between modules and consensus clusters was generated using WGCNA functions to show the Pearson correlation coefficient and *p*-values. The gene set analysis (GSA) was performed for each module against the HALLMARK data set (gsea-msigdb.org) using the R package OmicPath (https://github.com/CBIIT-CGBB/OmicPath). The top GSA hit was selected to name the module. The genes in each module were compared with every module in the CPTAC ovarian dataset (Zhang et al.^8^) to obtain the overlapping gene counts and calculate the percentage of overlapping genes.

### HRD analysis using scarHRD and CHORD

To run scarHRD (version 0.1.1 downloaded from https://github.com/sztup/scarHRD), somatic copy number data was extracted for each tumor sample from Canvas outputs and used as input to the ‘scar_score’ function within the scarHRD package. Three frequency scores (loss of heterozygosity, large scale transitions, and telomeric allelic imbalances) are reported for each sample, with the sum of these (“HRD-sum”) representing an overall HRD sample score.

To run CHORD (downloaded from https://github.com/UMCUGenetics/CHORD), somatic SNV + indel and structural variant (SV) VCF files were filtered for passing variants and supplied to the “extractSigsChord” function with the sv.caller parameter set to “manta”. The output from this function was then supplied to the “chordPredict” function with bootstrapping enabled. Default classifier-predicted HR status (“HR_proficient” or “HR_deficient”) were used for discrete classifications, while probabilities of HRD were used for statistical classifications.

### Neoepitope prediction from WGS and RNA-seq data

To predict neoepitopes in tumor specimens, we typed the HLA class I alleles for each tumor using the OptiType^59^ pipeline. Within the pipeline, RazerS3 was run using the following parameters: –percent-identity 95, –max-hits 1, –distance-range 0. Otherwise, default parameters were applied throughout the pipeline. For the resultant six HLA-A/B/C alleles, we utilized the default pVACseq pipeline to create a list of stringently filtered neoepitopes^60^ using MHCflurry, MHCnuggetsI, MHCnuggetsII, NNalign, NetMHC, PickPocket, SMM, SMMPMBEC, and SMMaligndefault as the epitope prediction algorithms, and otherwise used default parameters. Detailed parameters and pipeline scripts are described in https://github.com/shahcompbio/pvacseq_pipeline.

### Variant discovery in RNA-seq

To investigate if the neoepitope candidates uniquely predicted in ET (versus BT) are also observable in the companion RNA-seq data, we used the GATK best practice workflow for RNA-seq short variant discovery (https://gatk.broadinstitute.org/hc/en-us/articles/360035531192-RNAseq-short-variant-discovery-SNPs-Indels-). Reads were first aligned using the STAR aligner. The aligned reads underwent duplicate removal, CIGAR annotation-based read splitting, base recalibration, and finally variant calling using GATK. Read count annotation per variant was performed using Vt package and VCF Readcount Annotator. Detailed parameters and pipeline scripts are available in https://github.com/shahcompbio/rnaseq_variant_discovery. When applying the downstream RNA-seq variant support filter for neoepitope candidates, we selected the neoepitopes that had been discovered in this RNA-seq variant discovery pipeline

### Investigation of BMI1 inhibition in isogenic cell line models of homologous recombination deficient (HRD, UWB1.289) or HR proficient (UWB1.289 + BRCA1) high-grade serous ovarian cancer

UWB1.289 (CRL-2945) and UWB1.289 + BRCA1 (CRL-2946) cells were purchased from ATCC (Manassas, VA) and maintained in 50% RPMI (ATCC), 50% complete MEGM (Lonza, Walkersville, MD), 3% fetal bovine serum (ATCC) and 1X penicillin streptomycin (Thermo Fisher Scientific, Inc.). UWB1.289 + BRCA1 cells were maintained in G418 (200 µg/mL). Immunoblot analysis was performed as previously described^61^, where equivalent amounts of cell lysates generated from sub-confluent cultures were resolved by 1D gel electrophoresis (BioRad) and transferred to polyvinylidene difluoride membranes (BioRad), blocked in 5% powdered milk, 1X Tris-buffered saline with 0.1% Tween® 20 Detergent (TBST), (BioRad), and probed with antibodies specific for BRCA1 (OP92-100UG, Sigma Aldrich, Burlington, MA, United States), BMI1 (#6964, Cell Signaling Technology, Danvers MA) or beta-Actin (# 3700, Cell Signaling Technology). Colony survival assays were conducted with equivalent numbers of UWB1.289 or UWB1.289 + BRCA1 cells plated in six-well plates on day 1, and treatment with drug vehicle (DMSO), PTC-028 (#S8662, Selleckchem, Houston, TX) or PTC596 (# S8820, Selleckchem) on day 2. Cultures were maintained for ~7 days before being stained with crystal violet^62^ and counted. Three independent biological replicates of colony survival assays were performed for UWB1.289 and UWB1.289 + BRCA1 cells treated with PTC-028 or PTC596 and each condition was assessed as a technical replicate for each biological replicate assay.

### Reporting summary

Further information on research design is available in the Nature Research Reporting Summary linked to this article.

### Supplementary information

REPORTING SUMMARY

Supplementary Information

SupplementalDataDictionary

SupplementalData1

SupplementalData2

SupplementalData3

SupplementalData4

SupplementalData5

SupplementalData6

SupplementalData7

SupplementalData8

SupplementalData9

SupplementalData10

SupplementalData11

SupplementalData12

SupplementalData13

SupplementalData14

SupplementalData15

SupplementalData16

SupplementalData17

SupplementalData18

## Data Availability

Data generated in this study (DNA sequencing, mRNA sequencing, and proteomic data) are deposited at dbGap under study accession phs003488v1.p1; MS-based proteomics data are also available at the ProteomeXChange at PXD045417 and PXD045710. These data can also be interactively explored at www.lmdomics.org/APOLLO2. Further information and requests for resources and reagents should be directed to and will be fulfilled by the lead contacts, Nicholas W. Bateman (batemann@whirc.org), Thomas P. Conrads (conrads@whirc.org) or G. Larry Maxwell (George.Maxwell@inova.org).

## Code availability

Software code generated to support primary data analysis is available upon request.
