# Author Correction: Proteogenomic analysis of enriched HGSOC tumor epithelium identifies prognostic signatures and therapeutic vulnerabilities

**DOI:** 10.1038/s41698-024-00588-9

**Published:** 2024-05-06

**Authors:** Nicholas W. Bateman, Tamara Abulez, Anthony R. Soltis, Andrew McPherson, Seongmin Choi, Dale W. Garsed, Ahwan Pandey, Chunqiao Tian, Brian L. Hood, Kelly A. Conrads, Pang-ning Teng, Julie Oliver, Glenn Gist, Dave Mitchell, Tracy J. Litzi, Christopher M. Tarney, Barbara A. Crothers, Paulette Mhawech-Fauceglia, Clifton L. Dalgard, Matthew D. Wilkerson, Mariaelena Pierobon, Emanuel F. Petricoin, Chunhua Yan, Daoud Meerzaman, Clara Bodelon, Nicolas Wentzensen, Jerry S. H. Lee, The APOLLO Research Network, Sasha C. Makohon-Moore, Waleed Barakat, Xijun Zhang, Allison Hunt, Wei Ao, Stacey L. Lytle-Gabbin, Yovanni Casablanca, Chad A. Hamilton, Miranda Newell, Justin Wells, Gauthaman Sukumar, Dagmar Bacikova, John Freyman, David E. Cohn, Andrew Berchuck, Laura Havrilesky, Linda Duska, Adekunle Odunsi, Anil Sood, James Brenton, Evis Sala, Christina Annunziata, Oliver Dorigo, Brad Nelson, Dawn R. Cochrane, Kathleen Moore, Elisa Baldelli, Qing-rong Chen, Ying Hu, Sian Fereday, Nadia Traficante, Anna DeFazio, Ellen L. Goode, David G. Huntsman, Sohrab Shah, Craig D. Shriver, Neil T. Phippen, Kathleen M. Darcy, David D. L. Bowtell, Thomas P. Conrads, G. Larry Maxwell

**Affiliations:** 1 Gynecologic Cancer Center of Excellence, Gynecologic Surgery and Obstetrics, Uniformed Services University of the Health Sciences, Bethesda, MD USA; 2 The Henry M. Jackson Foundation for the Advancement of Military Medicine Inc, Bethesda, MD USA; 3 The John P. Murtha Cancer Center Research Program, Department of Surgery, Uniformed Services University and Walter Reed National Military Medical Center, Bethesda, MD USA; 4 The American Genome Center, Collaborative Health Initiative Research Program, Department of Anatomy, Physiology and Genetics, Uniformed Services University of the Health Sciences, Bethesda, MD USA; 5 Department of Computational Oncology, Memorial Sloan Kettering Cancer Center, Manhattan, NY USA; 6 Peter MacCallum Cancer Centre, Parkville, Melbourne, Victoria Australia; 7 Sir Peter MacCallum Department of Oncology, The University of Melbourne, Parkville, Victoria Australia; 8 The Joint Pathology Center, Defense Health Agency, National Capital Region Medical Directorate, Silver Spring, MD USA; 9 Department of Anatomic Pathology, Division of Gynecologic Pathology, University of Southern California, Los Angeles, CA USA; 10 Center for Applied Proteomics and Molecular Medicine, George Mason University, Manassas, VA USA; 11 Center for Biomedical Informatics and Information Technology, National Cancer Institute, Rockville, MD USA; 12 Division of Cancer Epidemiology and Genetics National Cancer Institute, Rockville, MD USA; 13 Ellison Institute for Transformative Medicine, University of Southern California, Los Angeles, CA USA; 14 Department of Pathology and Laboratory Medicine, The University of British Columbia, Vancouver, British Columbia Canada; 15 Women’s Health Integrated Research Center, Women’s Service Line, Inova Health System, Falls Church, VA USA; 16 The Cancer Imaging Archive, National Cancer Institute, Bethesda, MD USA; 17 The Ohio State University, Columbus, OH USA; 18 Duke University Medical Center, Durham, NC USA; 19 University of Virginia, Charlottesville, VA USA; 20 University of Chicago, Chicago, IL USA; 21 MD Anderson Comprehensive Cancer Center, Houston, TX USA; 22 University of Cambridge, Cambridge, UK; 23 National Cancer Institute, Bethesda, MD USA; 24 Stanford University, Stanford, CA USA; 25 British Columbia Cancer Research Centre, Vancouver, Canada; 26 University of Oklahoma Health Sciences Center, Oklahoma City, OK USA; 27 The Australian Ovarian Cancer Study Group, Peter MacCallum Cancer Centre, Melbourne, Victoria Australia; 28 The Westmead Institute for Medical Research, Sydney, NSW Australia; 29 Department of Gynaecological Oncology, Westmead Hospital, Sydney, NSW Australia; 30 The University of Sydney, Sydney, NSW Australia; 31 Mayo Clinic, Rochester, MN USA

**Keywords:** Ovarian cancer, Oncology

Correction to: *npj Precision Oncology* 10.1038/s41698-024-00519-8, published online 13 March 2024

In the original version of this article, the wrong file was inadvertently supplied for Supplementary Data 15. The original article has been corrected.

