# Supplementary material for: Proteogenomic analysis of enriched HGSOC tumor epithelium identifies prognostic signatures and therapeutic vulnerabilities
Source: NPJ Precis Oncol. 2024 Mar 13;8:68. doi: 10.1038/s41698-024-00519-8 (PMC10937683; doi:10.1038/s41698-024-00519-8)
Supplement: Supplementary file 1 — REPORTING SUMMARY [file 41698_2024_519_MOESM1_ESM.pdf]

nature portfolio

Corresponding author(s): Nicholas W. Bateman, Thomas P. Conrads, G. Larry Maxwell

Last updated by author(s): Dec 11, 2023

Reporting Summary

Nature Portfolio wishes to improve the reproducibility of the work that we publish. This form provides structure for consistency and transparency in reporting. For further information on Nature Portfolio policies, see our Editorial Policies and the Editorial Policy Checklist.

Statistics

For all statistical analyses, confirm that the following items are present in the figure legend, table legend, main text, or Methods section.

n/a	Confirmed	
☐	☒	The exact sample size (*n*) for each experimental group/condition, given as a discrete number and unit of measurement
☐	☒	A statement on whether measurements were taken from distinct samples or whether the same sample was measured repeatedly
☐	☒	The statistical test(s) used AND whether they are one- or two-sided<br>*Only common tests should be described solely by name; describe more complex techniques in the Methods section.*
☐	☒	A description of all covariates tested
☐	☒	A description of any assumptions or corrections, such as tests of normality and adjustment for multiple comparisons
☐	☒	A full description of the statistical parameters including central tendency (e.g. means) or other basic estimates (e.g. regression coefficient) AND variation (e.g. standard deviation) or associated estimates of uncertainty (e.g. confidence intervals)
☐	☒	For null hypothesis testing, the test statistic (e.g. *F*, *t*, *r*) with confidence intervals, effect sizes, degrees of freedom and *P* value noted<br>*Give P values as exact values whenever suitable.*
☒	☐	For Bayesian analysis, information on the choice of priors and Markov chain Monte Carlo settings
☐	☒	For hierarchical and complex designs, identification of the appropriate level for tests and full reporting of outcomes
☒	☐	Estimates of effect sizes (e.g. Cohen's *d*, Pearson's *r*), indicating how they were calculated

Our web collection on statistics for biologists contains articles on many of the points above.

Software and code

Policy information about availability of computer code

Data collection	Liquid chromatography-tandem mass spectrometry (LC-MS/MS) analyses of TMT-11 multiplexes was performed on a nanoflow high-performance LC system (EASY-nLC 1200, Thermo Fisher Scientific) connected online with an Orbitrap mass spectrometer (Q Exactive HF-X, Thermo Fisher Scientific).<br><br>RPPA was performed by printing tissue lysates derived from LMD onto nitrocellulose coated slides (Grace Bio-labs, Bend, OR) using an Aushon 2470 arrayer (Aushon BioSystems, Billerica, MA).<br><br>Methylation was measured using the Infinium MethylationEPIC BeadChip (Illumina Inc.).<br><br>Clustering and RNA sequencing were performed on the HiSeq 500 (Illumina) using a High Output 150 cycle kit for paired-end reads of 75 bp length and an intended depth of 50 million reads per sample.<br><br>Paired-end sequencing was performed with the HiSeq X HD SBS Kit (300 cycles) on the Illumina HiSeq X.
Data analysis	Mass spectrometry data files were searched against a publicly available, non-redundant human proteome database (Swiss-Prot, Homo sapiens, http://www.uniprot.org) using Mascot (Matrix Science, Boston, MA, USA) and Proteome Discoverer (Thermo Fisher Scientific).<br><br>RPPA images were analyzed with a commercially available software (MicroVigene 5.1.0.0; Vigenetech, Carlisle, MA).<br><br>Methylation array raw data files (idat files) were processed with the minfi R package.

Differential analyses of global proteome or transcriptome matrixes were performed using the LIMMA package (version 3.8) in R (version 3.5.2). Pathway analysis was performed using Metascape (https://metascape.org/gp/index.html#/main/step) using default parameters. Molecular subtype analysis was generated by consensusOV (version 1.12.0) from whole and enriched transcript matrices ad transitions were plotted as a Sankey plot with networkD3 (version 0.4) in R Studio (version 3.6.0).

The sparse Partial Least Squares Discriminant Analysis (sPLS-DA) model was first optimized on a 70:30 percent split of the transcript data to optimize the number of components selected for the final model (mixOmics version 6.8.5; caret version 6.0-86).

The co-expression network was constructed through the "WGCNA" package (version 1.69) in the R environment (version 3.6.2).

The gene set analysis (GSA) was performed for each module against HALLMARK data set (gsea-msigdb.org) using the R package OmicPath (https://github.com/CBIIT-CGBB/OmicPath).

To run scarHRD (version 0.1.1), somatic copy number data was extracted for each tumor sample from Canvas outputs and used as input to the 'scar_score' function within the scarHRD package.

To predict the neoepitopes per tumor specimen, we first typed the HLA class I alleles for each tumor using the OptiType62 pipeline. Within the pipeline, RazerS3 was run using the following parameters: --percent-identity 95, --max-hits 1, --distance-range 0. Otherwise default parameters were applied throughout the pipeline. For the resultant six HLA-A/B/C alleles, we utilized the default pVACseq pipeline to create a list of stringently filtered neoepitopes63 using MHCflurry, MHCnuggetsI, MHCnuggetsII, NNalign, NetMHC, PickPocket, SMM, SMMPMBEC, and SMMaligndefault as the epitope prediction algorithms, and otherwise used default parameters. Detailed parameters and pipeline scripts are described in https://github.com/shahcompbio/pvacseq_pipeline.

Transcript expression levels were quantified using the tool of Kallisto "quant"64, using GRCh38 GTF as gene annotations, and otherwise default parameters except for "--bootstrap-samples 2". When applying the downstream expression filters for neoepitope candidates, we set the expression cutoff to Kallisto TPM (Transcripts Per Million) of 1.0.

To investigate if the neoepitope candidates uniquely predicted in enriched tumors (versus bulk tumors) are also observable in the companion RNA-seq data, we used the GATK best practice workflow for RNA-seq short variant discovery (https://gatk.broadinstitute.org/hc/en-us/articles/360035531192-RNAseq-short-variant-discovery-SNPs-Indels-).

For manuscripts utilizing custom algorithms or software that are central to the research but not yet described in published literature, software must be made available to editors and reviewers. We strongly encourage code deposition in a community repository (e.g. GitHub). See the Nature Portfolio guidelines for submitting code & software for further information.

Data

Policy information about availability of data

All manuscripts must include a data availability statement. This statement should provide the following information, where applicable:

- Accession codes, unique identifiers, or web links for publicly available datasets
- A description of any restrictions on data availability
- For clinical datasets or third party data, please ensure that the statement adheres to our policy

Data generated in this study (DNA sequencing, mRNA sequencing, and proteomic data) are deposited at dbGap under study accession phs003488v1.p1; MS-based proteomics data are also available at the ProteomeXChange at PXD045417. These data can also be interactively explored at www.lmdomics.org/APOLLO2. Further information and requests for resources and reagents should be directed to and will be fulfilled by the lead contacts, Nicholas W. Bateman (batemann@whirc.org), Thomas P. Conrads (conrads@whirc.org) or G. Larry Maxwell (Larry Maxwell (George.Maxwell@inova.org).

Research involving human participants, their data, or biological material

Policy information about studies with human participants or human data. See also policy information about sex, gender (identity/presentation), and sexual orientation and race, ethnicity and racism.

Reporting on sex and gender	Due to the nature of ovarian cancer, all patients included in this study were female.
Reporting on race, ethnicity, or other socially relevant groupings	Race and ethnicity (table 1) are reported as self-described.
Population characteristics	Relevant population characteristics for ovarian cancer were utilized in the multivariate analyses employed, and included age, stage, and debulking status.
Recruitment	Patients were recruited onto the IRB-approved protocol by gynecologic oncology providers in the clinic.
Ethics oversight	Patients were enrolled in the WCG IRB approved #20110222 Tissue and Data Acquisition Study of Gynecologic Disease who underwent primary debulking surgery or a diagnostic laparoscopy at Inova Fairfax Medical Campus (Inova), Duke University Medical Center (Duke) or the Ohio State University (OSU); written informed consent was obtained from all subjects involved in the study.

Note that full information on the approval of the study protocol must also be provided in the manuscript.

Field-specific reporting

Please select the one below that is the best fit for your research. If you are not sure, read the appropriate sections before making your selection.

☒ Life sciences ☐ Behavioural & social sciences ☐ Ecological, evolutionary & environmental sciences

For a reference copy of the document with all sections, see nature.com/documents/nr-reporting-summary-flat.pdf

Life sciences study design

All studies must disclose on these points even when the disclosure is negative.

Sample size	No sample size calculations were performed. The sample size was determined pragmatically based on the extensive number and cost of multi-omics analyses performed on each tissue specimen, which included WGS, RNA-seq, methylation sequencing, RPPA, and mass spectrometry-proteomics on bulk tissue, laser microdissected tumor epithelium, and laser microdissected stroma (for a subset of cases).
Data exclusions	No data were excluded from the analysis.
Replication	Due to the high cost of multi-omics analyses, WGS, RNA-sequencing, methylation arrays, RPPA, and mass spectrometry-based proteomics were prformed only once for each biochemical specimen generated from each tissue. Multiple publicly available data sets (Hunt et al 2021, Zhang et al 2016, TGCA 2011, Garsed et al 2022, Zhang et al 2018) were used for validation of multiple mutational and expression signatures described in the present work.
Randomization	All samples were randomized prior to generating the omics data.
Blinding	All analysts involved in generating data were blinded.

Reporting for specific materials, systems and methods

We require information from authors about some types of materials, experimental systems and methods used in many studies. Here, indicate whether each material, system or method listed is relevant to your study. If you are not sure if a list item applies to your research, read the appropriate section before selecting a response.

Materials & experimental systems

n/a	Involved in the study	
☐	☒	Antibodies
☐	☒	Eukaryotic cell lines
☒	☐	Palaeontology and archaeology
☒	☐	Animals and other organisms
☒	☐	Clinical data
☒	☐	Dual use research of concern
☒	☐	Plants

Methods

n/a	Involved in the study	
☒	☐	ChIP-seq
☒	☐	Flow cytometry
☒	☐	MRI-based neuroimaging

Antibodies

Antibodies used	Antibodies specific for BRCA1 (OP92-100UG, Sigma Aldrich, Burlington, MA, United States), BMI1 (#6964, Cell Signaling Technology, Danvers MA, USA) or beta-Actin (# 3700, Cell Signaling Technology, Danvers MA, USA) were used.
Validation	Validation involved demonstrating a single band on the western blot for the cell lines probed.

Eukaryotic cell lines

Policy information about cell lines and Sex and Gender in Research

Cell line source(s)	UWB1.289 (CRL-2945) and UWB1.289 + BRCA1 (CRL-2946) cells were purchased from ATCC (Gaithersburg, MD USA).
Authentication	Cell lines were purchased as authenticated.
Mycoplasma contamination	All cell lines tested negative for mycoplasma contamination.
Commonly misidentified lines (See ICLAC register)	not applicable
