# Supplementary material for: Proteogenomic analysis of enriched HGSOC tumor epithelium identifies prognostic signatures and therapeutic vulnerabilities
Source: NPJ Precis Oncol. 2024 Mar 13;8:68. doi: 10.1038/s41698-024-00519-8 (PMC10937683; doi:10.1038/s41698-024-00519-8)
Supplement: Supplementary file 2 — Supplementary Information [file 41698_2024_519_MOESM2_ESM.pdf]

Proteogenomic Analysis of Enriched HGSOC Tumor Epithelium Identifies Prognostic Signatures and Therapeutic Vulnerabilities

Supplementary Information

Extended Data Supplement

Somatic mutation analysis identified tumor protein 53 (*TP53*) as the top significantly altered gene in our APOLLO-2 cohort (Fig. 1b). Notably, we observed mutations in *TP53* in bulk tissue (BT) collections except for case A126 where we identified a "likely pathogenic" mutation in *TP53* (rs730881999) in WGS data derived from companion enriched tumor (ET) collections. Analysis of somatic SNV signatures showed subset tumors are also enriched for aging, single-base substitution 5 (SBS5) as well as SBS40 SNV signatures (Clusters 4 and 5, Fig. 1b, Supplementary Table 2), consistent with recent reports showing that SBS5 and SBS40 correlate with aging signatures in cancer[1, 2, 3]. We also identified patient tumors enriched for HRD and SBS8 SNV signatures (Cluster 3, Fig. 1b, Supplementary Table 2), with many of these tumors also harboring germline or somatic mutations in breast cancer type 1 or 2 susceptibility genes (odds ratio = 17.9, 4.8-67, $p < 0.0001$, Fig. 1b). Most tumors with HRD SNV signatures did not harbor amplifications in cyclin E (*CCNE1*); tumors classified as HRD-duplicated or HRD-deleted did have significantly increased somatic SNV mutations (Mann Whitney U, MWU, $p \leq 0.0012$) and somatic deletions (MWU, $p \leq 0.0003$) compared with tumors with low HRD signatures (Supplementary Table 2), consistent with recent evidence reported in HGSOC[4]. In addition, tumors with HRD and SBS8 SNV signatures were also enriched for short (S-Dup) and middle (M-Dup) duplication events (Cluster 3, Fig. 1b), which have previously been shown to co-occur in breast and ovarian cancers[5]. Comparison of SV signatures identified from bulk tumor (BT) whole genome sequencing (WGS) data in the APOLLO-2 cohort with a recently described "metacohort" of 170 HGSOC tumors[5] showed high correlation of SV subtype distributions, with most tumors classified as having fold-back inversions (FBI), followed by HRD-deleted (HRD-Del), HRD-duplicated (HRD-Dup) and tandem duplicated (TD) subtypes (Supplementary Table 2). We also find that patient tumors classified as HRD-Dup have superior outcomes relative to FBI tumors (Supplementary Fig. 1a), also consistent with recent reports [5, 6].

References

1. Singh VK, Rastogi A, Hu X, Wang Y, De S. Mutational signature SBS8 predominantly arises due to late replication errors in cancer. *Commun Biol* **3**, 421 (2020).
2. Heredia-Genestar JM, Marques-Bonet T, Juan D, Navarro A. Extreme differences between human germline and tumor mutation densities are driven by ancestral human-specific deviations. *Nat Commun* **11**, 2512 (2020).
3. Alexandrov LB*, et al.* The repertoire of mutational signatures in human cancer. *Nature* **578**, 94-101 (2020).
4. Takaya H, Nakai H, Takamatsu S, Mandai M, Matsumura N. Homologous recombination deficiency status-based classification of high-grade serous ovarian carcinoma. *Sci Rep* **10**, 2757 (2020).
5. Funnell T*, et al.* Integrated structural variation and point mutation signatures in cancer genomes using correlated topic models. *PLoS Comput Biol* **15**, e1006799 (2019).

6. Wang YK*, et al.* Genomic consequences of aberrant DNA repair mechanisms stratify ovarian cancer histotypes. *Nat Genet* **49**, 856-865 (2017).
7. Zhang AW*, et al.* Interfaces of Malignant and Immunologic Clonal Dynamics in Ovarian Cancer. *Cell* **173**, 1755-1769 e1722 (2018).
8. Garsed DW*, et al.* The genomic and immune landscape of long-term survivors of high-grade serous ovarian cancer. *Nature Genetics, Accepted July 11th, 2022*, (2022).
9. Cancer Genome Atlas Research N. Integrated genomic analyses of ovarian carcinoma. *Nature* **474**, 609-615 (2011).

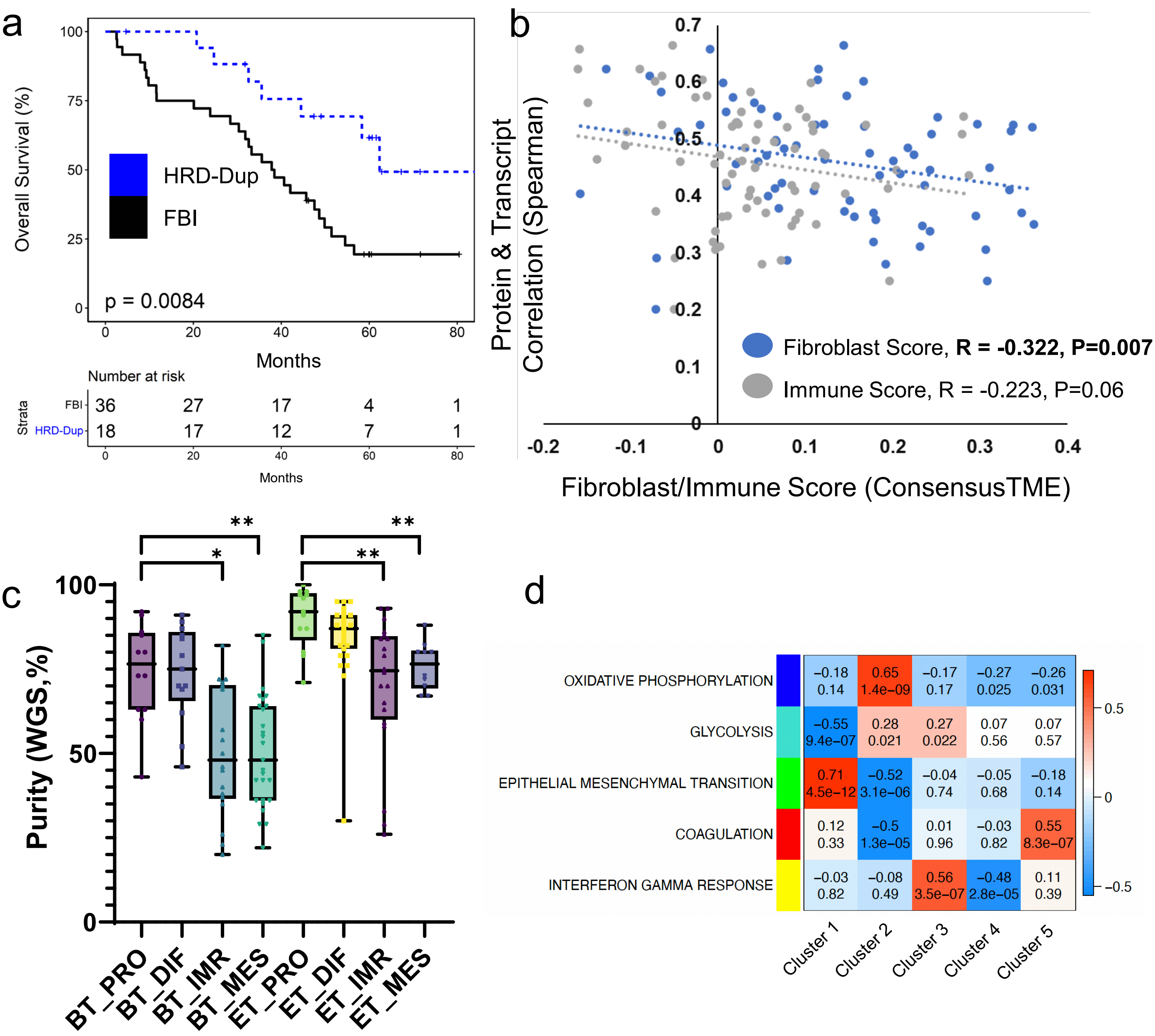

e

APOLLO-2

CPTAC 2016

Modules	Coagulation	Epithelial Mesenchymal Transition	Glycolysis	Interferon Gamma Response	Oxidative Phosphorylation
cell-cell communications	0	1.36	0.31	0.1	1.99
complement cascade	7.95	1.15	1.05	0.52	0.31
cytokine signaling	0.73	0	1.67	12.03	0.73
DNA replication	1.57	0.52	3.35	0.1	0.63
ECM interaction	1.36	16.95	2.41	1.67	0.21
erythrocyte and platelet	2.41	0.1	0.84	0	0
metabolism	1.99	1.36	18.62	1.26	14.75

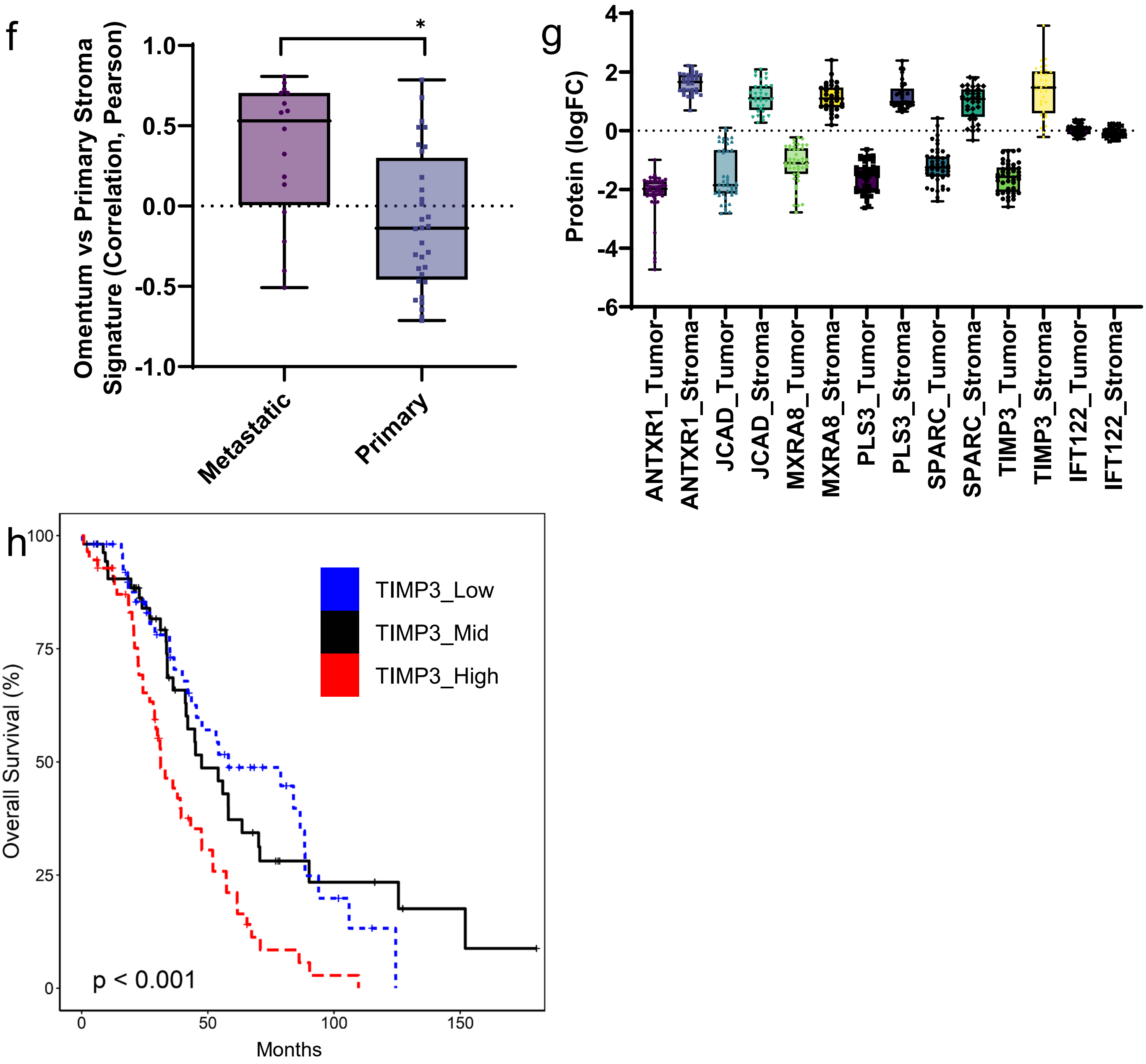

Supplementary Fig. 1 | See next page for caption.

Supplementary Figure 1. a. Kaplan-Meier overall survival curves for patients whose tumors were characterized as HR-Duplicated (n = 18, HRD-Dup, blue dotted line) or having a foldback inversion signature (n = 36, FBI, black line, *p* value reflects log rank testing). b. Correlation of sample-wise protein and transcript correlations with immune or fibroblast scores (ConsensusTME) calculated using bulk tumor (BT) derived transcriptome data for 70 patient tumors. c. Comparison of tumor purity estimates derived from whole genome sequencing data by Consensus OV molecular subtype (DIF, differentiated; IMR, immunoreactive; MES, mesenchymal; PRO, proliferative) from BT and laser microdissection enriched tumor (ET) collections (Mann-Whitney U (MWU) $p < 0.05$, **$p < 1E\text{-}2$)). d. Heatmap integrating weighted gene co-expression network analysis (WGCNA) modules from five protein consensus clusters from BT data and HALLMARK molecular pathways enriched (gsea-msigdb.org) in BT data further correlated with consensus cluster designations. e. Confusion matrix representing proteins overlapping with WGCNA modules described in Supplementary Fig. 1d with HALLMARK pathways enriched within a WGCNA analysis for an independent cohort of CPTAC HGSOC tumors. f. Correlation of 47 stroma signature proteins from Eckert *et al.* quantified in enriched stroma (ES) from APOLLO-2 adnexal (n = 32) or metastatic (n = 16) tumors (MWU $p = 0.0025$). G. Relative abundance of proteins in HGSOC tumor epithelium and stroma identified from Hunt *et al* as significantly altered between MES vs DIF & PRO tumors that correlate with overall survival; DYNC2LI1 was not quantified by Hunt *et al*. h. Kaplan-Meier overall survival curves for tertiles of metalloproteinase inhibitor 3 (TIMP3) abundance in CPTAC HGSOC patients (n = 163) (Log Rank, $p < 0.0001$).

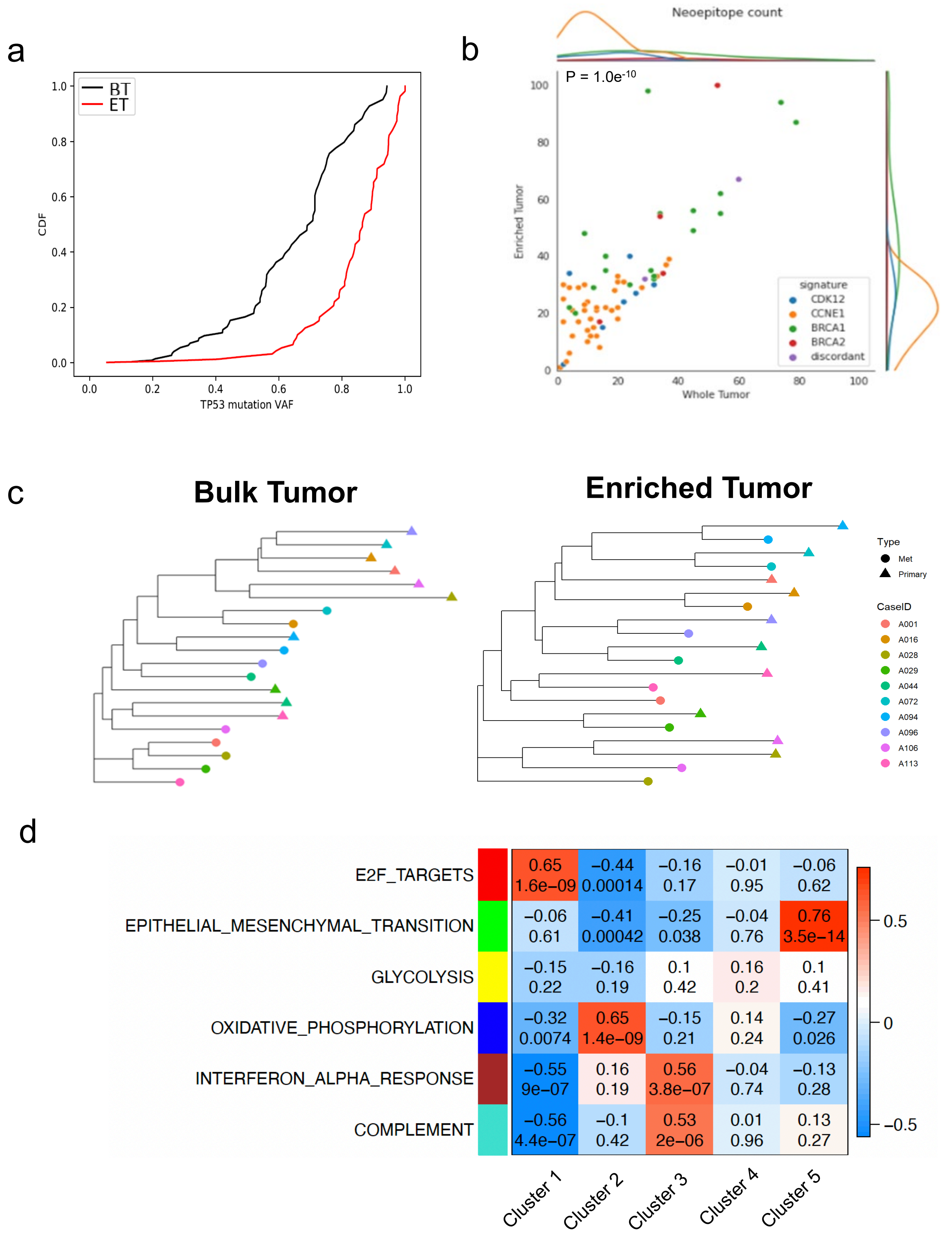

Supplementary Figure 2 | See next page for caption.

Supplementary Figure 2. a. Cumulative distribution frequency plot comparing variable allele frequencies (VAFs) for *TP53* determined from whole genome sequencing analysis of bulk tumor (BT) or laser microdissection enriched tumor (ET) collections (paired t test, p = 9.3e-17). b. Comparison of neoepitope counts predicted from WGS data in ET versus BT collections further stratified by *CCNE1* amplification or mutations in *CDK12*, *BRCA1* or *BRCA2* genes (Wilcoxon signed-rank test, $p = 1e^{-10}$). c. Dendrograms illustrating the relatedness between matched adnexal and metastatic tumors for 10 patients in BT and ET collections based on the most variably abundant proteins quantified in these samples (MAD > 0.75). The average distance in BT (0.65) versus ET (0.51) are significantly different (paired t-test, p = 0.002). d. Heatmap integrating weighted gene co-expression network analysis modules calculated using the top 25% most variably abundant proteins from ET data and HALLMARK molecular pathways enriched in these clusters further correlated with consensus clusters 1-5.

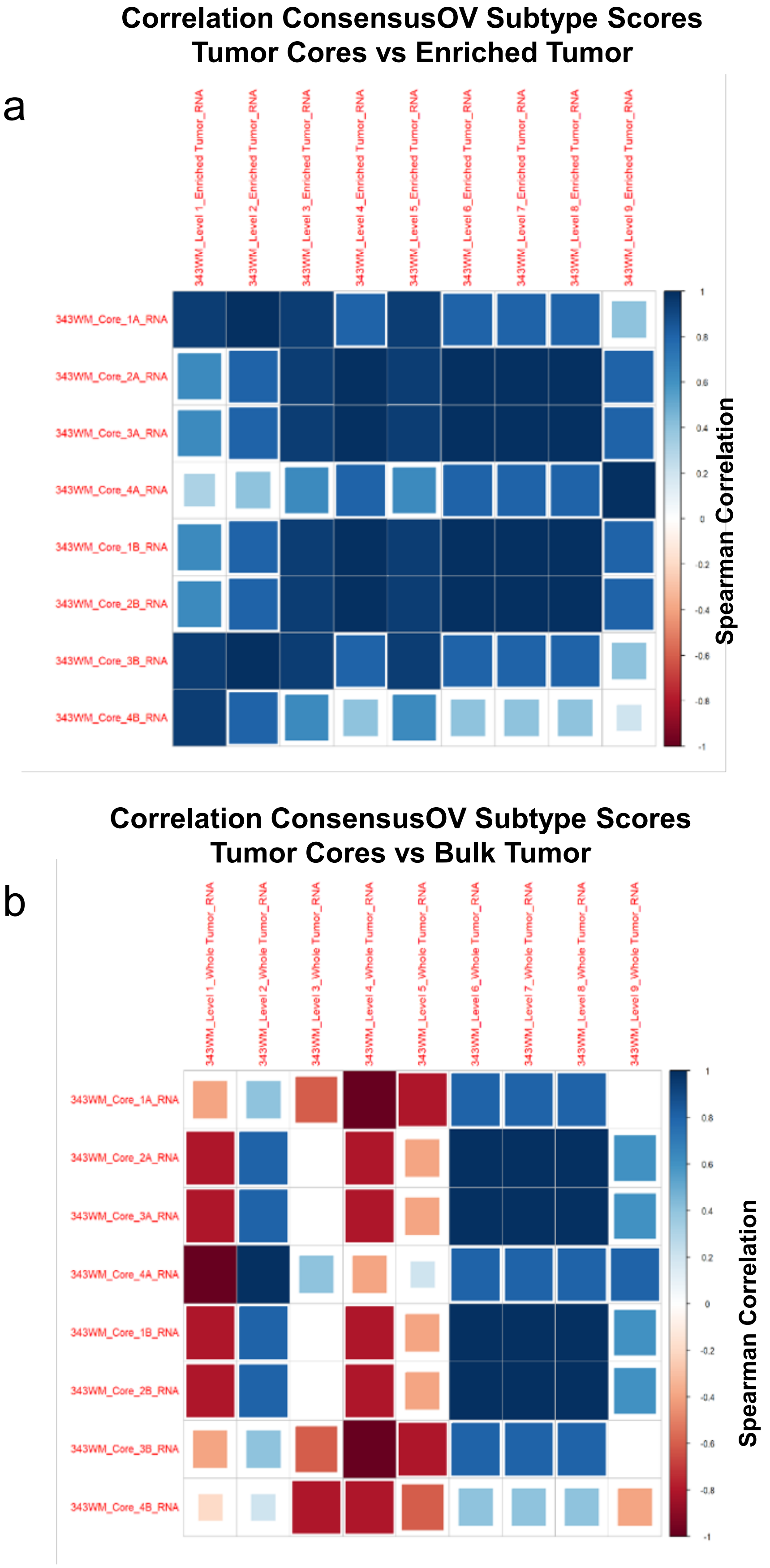

Supplementary Fig. 3 | See next page for caption.

Supplementary Figure 3. Correlation of ConsensusOV subtype scores adapted from Figure 5A in Hunt *et al*, 2021. a. Heatmap of Spearman correlation scores comparing ConsensusOV subtypes classified using tumor cores vs enriched tumor collections (average Spearman Rho = 0.81 ± 0.21) and b. heatmap of Spearman correlation scores comparing ConsensusOV subtypes classified using tumor cores vs whole (bulk) tumor collections (average Spearman Rho = 0.161 ± 0.71, MWU p < 1E-4).

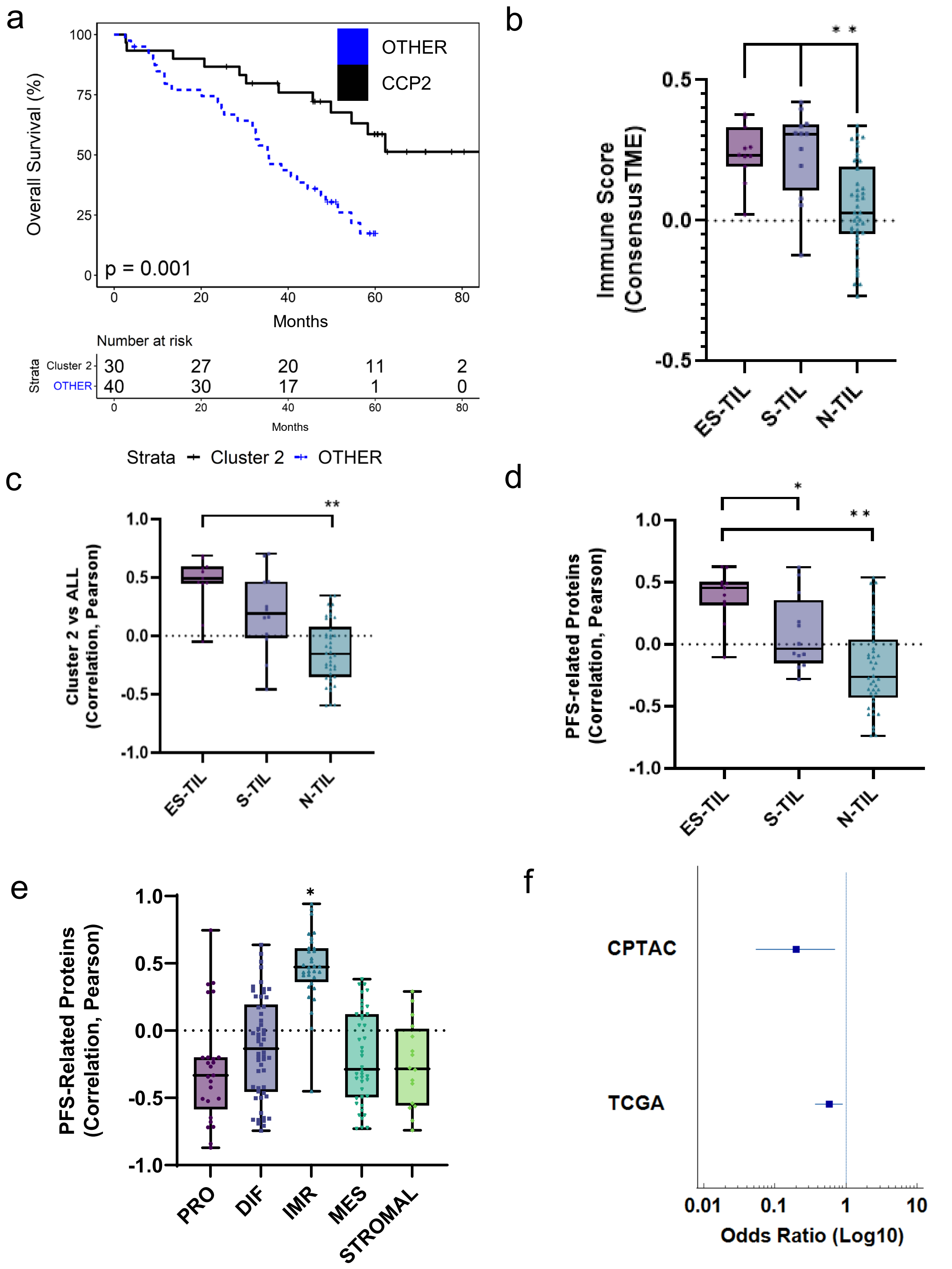

Supplementary Fig. 4 | See next page for caption.

Supplementary Figure 4. a. Kaplan-Meier overall survival curves for patients in consensus cluster 2 (CCP2) compared with those in CCP1/3/4 (OTHER) in Fig. 3a (*p*-value determined by a log rank test). b. Immune scores (ConsensusTME) derived from BT proteomics data from representative specimens from the Zhang *et al.* cohort in major tumor infiltrating lymphocyte (TIL) high grade serous ovarian cancer subtypes: ES-TIL (tumors with substantial levels of both epithelial and stromal TILs, n = 11) S-TIL (tumors dominated by stromal TILs, n = 12), and N-TIL (tumors sparsely infiltrated by TILs n = 42), **N-TIL vs ES-TIL/ S-TIL MWU $p \leq$ 7E-4. c. 163 altered proteins in CCP2 vs CCP1/3/4 cases are most strongly correlated with ES-TIL (n = 11 tumor samples), followed by S-TIL tumors (n = 12), and least correlated with N-TIL tumors (n = 42) in the Zhang *et al* HGSOC cohort (** denotes MWU U, $p < 0.0001$). d. Correlation of 15 significantly altered proteins from CCP2 vs CCP1/3/4 correlated with PFS (multivariate continuous Cox Chi-Square, $p < 0.05$) with global proteomic data generated from tissues reported in Zhang *et al.* are most correlated with ES-TIL, followed by S-TIL and least with N-TIL tumors (* denotes MWU, $p = 0.0035$, ** $p < 0.0001$). e. Fifteen progression-free survival related proteins are significantly correlated (*MWU, $p < 0.0001$) with tumors classified as immunoreactive (IMR) in the CPTAC HGSOC cohort (n = 169). (PRO, proliferative; DIF, differentiated; MES, mesenchymal). f. Odds ratio analysis comparing risk of death in patient tumors correlating with a 15 prognostic protein signature in CPTAC or TCGA cases (data reflects odds ratio < 1, Fisher's Exact $p < 0.05$ for both cohorts).

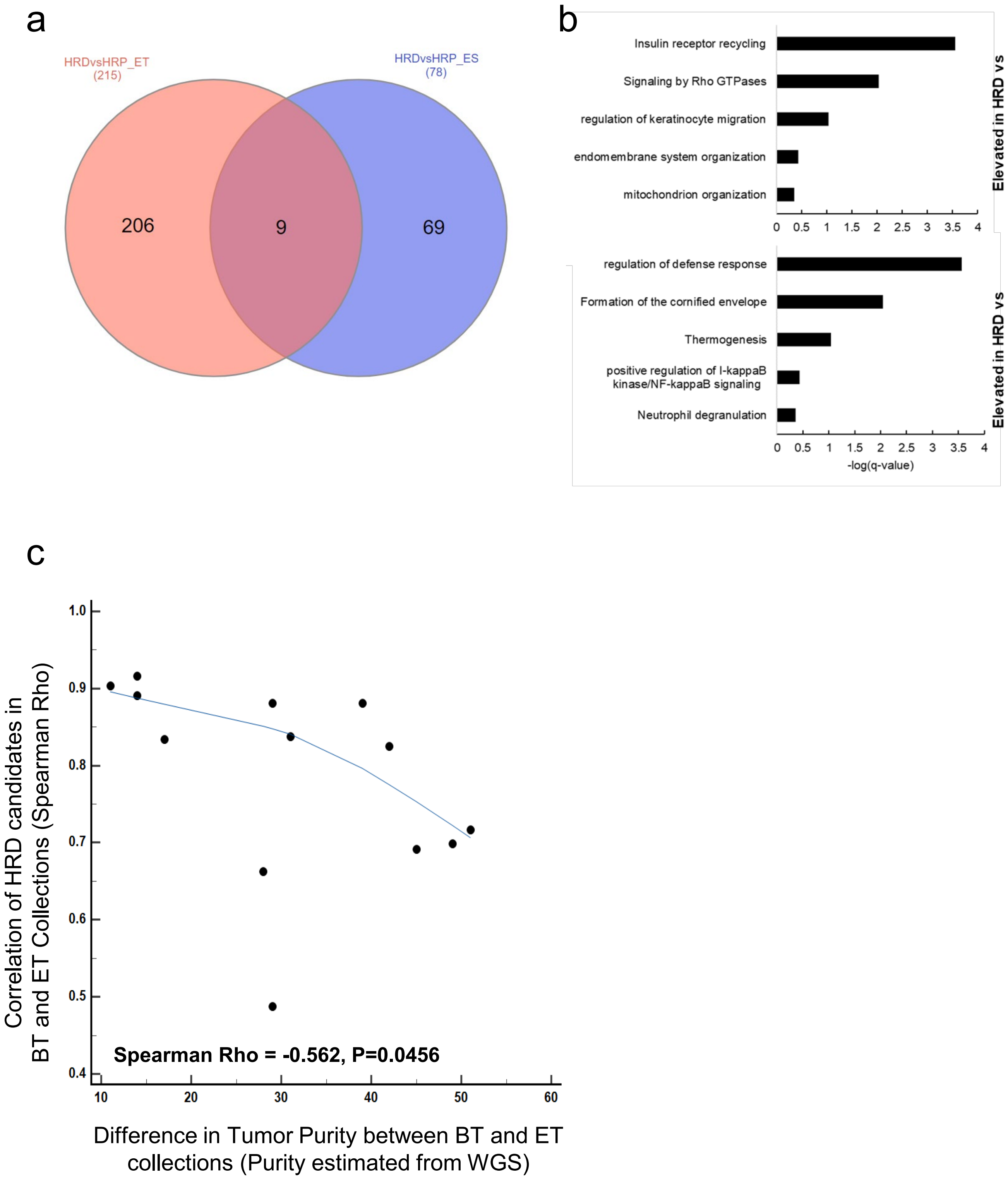

Supplementary Fig. 5 | See next page for caption.

Supplementary Figure 5. Comparison of protein alterations in enriched tumor (ET) or enriched stroma (ES) collections for homologous recombination deficient (HRD, n=13) and HR proficient (HRP, n=35) tumors. a. Comparison of proteins significantly altered between HRD vs HRP tumors in ET (LIMMA $p < 0.01$) or ES (LIMMA $p < 0.05$) collections. b. Top 5 pathways enriched among proteins elevated in HRD tumors in ET or ES collections (metascape.org). c. Correlation of protein abundances for candidates altered between HRD versus HRP tumors in BT and ET proteome data compared with differences in tumor purity estimates calculated from companion WGS data (Spearman Rho = -0.562, $p = 0.0456$).

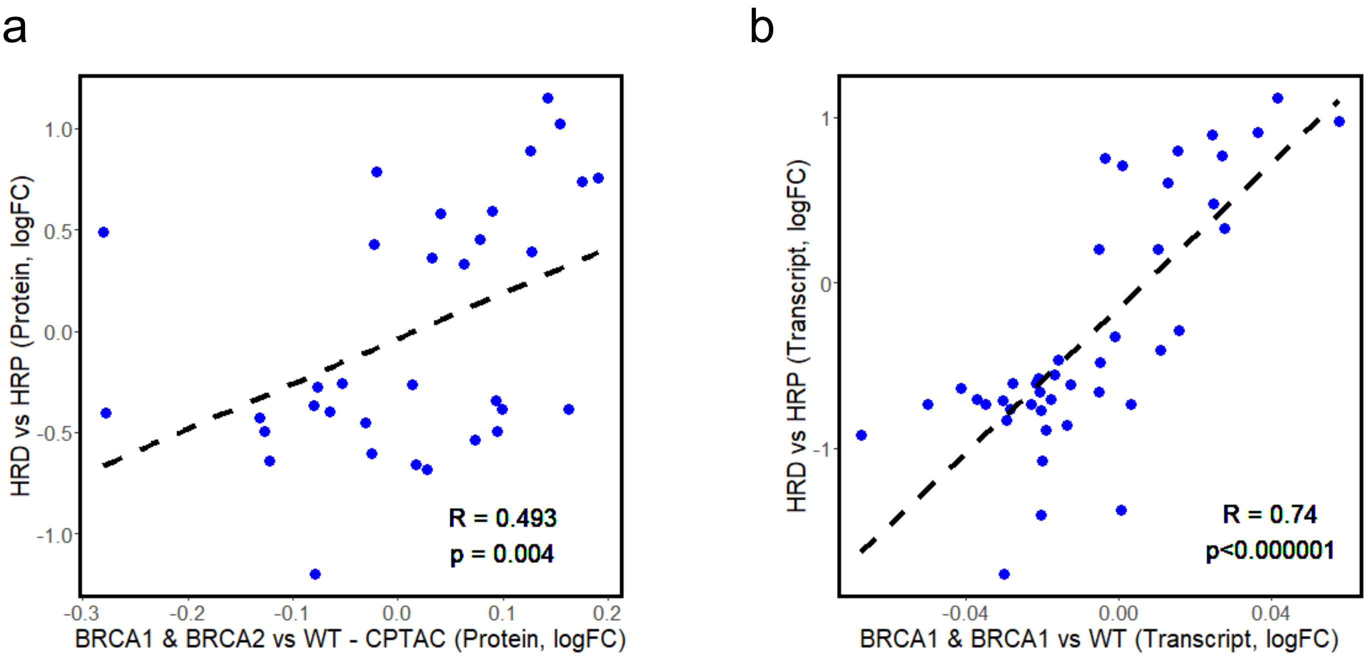

Supplementary Fig. 6 | See next page for caption.

Supplementary Figure 6. a. Correlation analysis of 33 HRD associated proteins in *BRCA1* and *BRCA2* mutant (n = 15) and wildtype (n = 125) HGSOC tumors from CPTAC HGSOC cohort. b. Correlation analysis of 43 HRD related transcripts mapping to *BRCA1/2* mutant (n = 67) vs wildtype (WT) (n = 422) HGSOC tumors from the TCGA cohort.

a

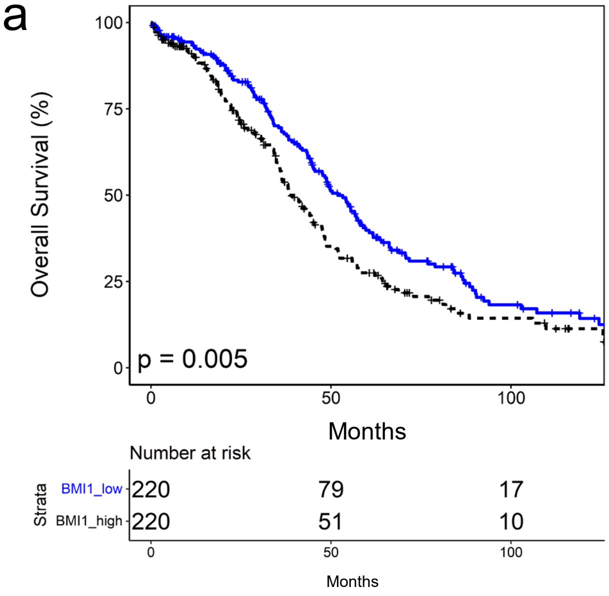

b

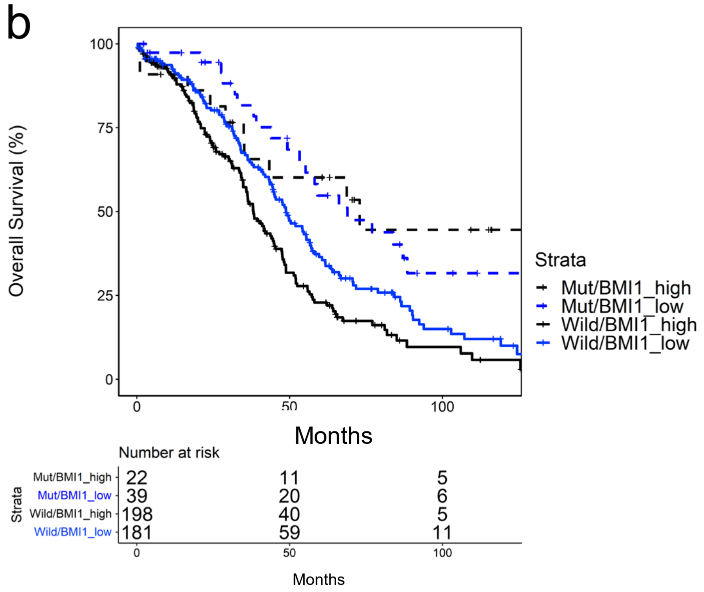

c

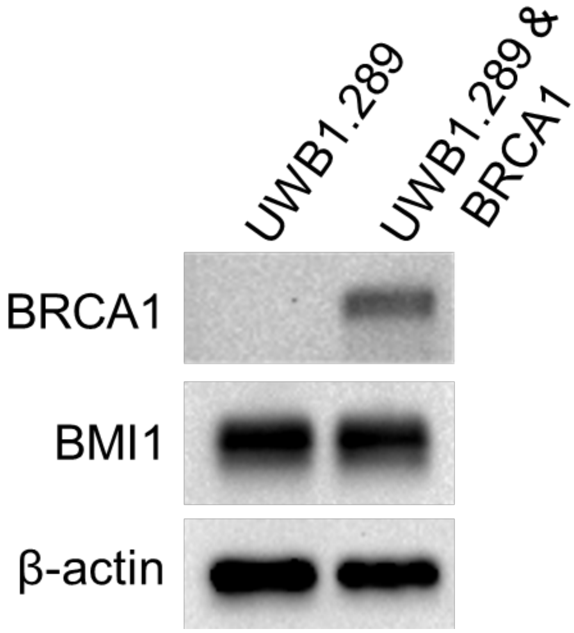

Supplementary Fig. 7 | See next page for caption.

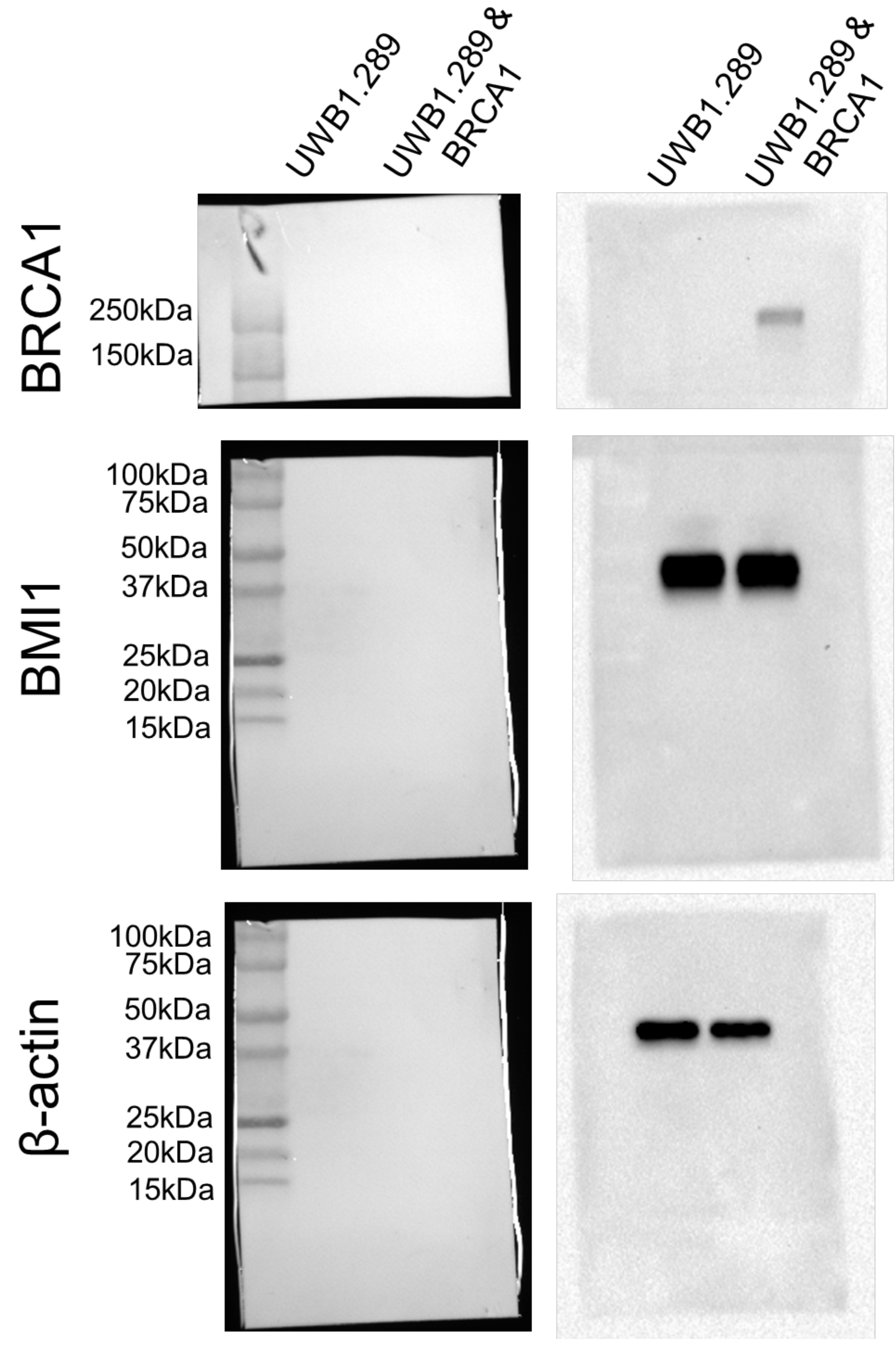
UWB1.289
UWB1.289 & BRCA1
UWB1.289
UWB1.289 & BRCA1
BRCA1
250kDa
150kDa
BMI1
100kDa
75kDa
50kDa
37kDa
25kDa
20kDa
15kDa
β-actin
100kDa
75kDa
50kDa
37kDa
25kDa
20kDa
15kDa

Supplementary Figure 7. a. Overall survival of HGSOC patients from the TCGA HGSOC cohort with tumors with elevated (BMI1_high, n=220) versus decreased (BMI1_low, n=220) levels of BMI1 transcript abundance, *p*-value reflects Log Rank testing. b. Overall survival of HGSOC patients with tumors exhibiting elevated (BMI1_high) versus decreased (BMI1_low) levels of BMI1 transcript abundance further stratified by patients with (n=61, multivariate $p = 0.997$, log rank $p = 0.84$) or without (n=379, multivariate *p*-value = 0.019, log rank $p = 0.004$) mutations in *BRCA1* or *BRCA2* (Supplementary Table 16). c. Immunoblot analysis of BMI1 and BRCA1 protein abundance in an isogenic cell line model of HRD (UWB1.289) and HRP (UWB1.289 + BRCA1) HGSOC cells. All blots were from the same experiment and processed in parallel.
